# Valorisation of Three Underutilised Native Australian Plants: Phenolic and Organic Acid Profiles and In Vitro Antimicrobial Activity

**DOI:** 10.3390/foods12030623

**Published:** 2023-02-01

**Authors:** Maral Seididamyeh, Anh Dao Thi Phan, Dharini Sivakumar, Michael E. Netzel, Ram Mereddy, Yasmina Sultanbawa

**Affiliations:** 1 Centre for Nutrition and Food Sciences, Queensland Alliance for Agriculture and Food Innovation, The University of Queensland, St Lucia, QLD 4072, Australia; 2 ARC Industrial Transformation Training Centre for Uniquely Australian Foods, Queensland Alliance for Agriculture and Food Innovation, The University of Queensland, Indooroopilly, QLD 4068, Australia; 3 Phytochemical Food Network Research Group, Department of Crop Sciences, Tshwane University of Technology, Pretoria West 0001, South Africa; 4 Department of Agriculture and Fisheries, Queensland Government, Coopers Plains, QLD 4108, Australia

**Keywords:** *Diploglottis bracteata*, *Syzygium aqueum*, *Tasmannia lanceolata*, antimicrobial activity, indigenous plant extracts, phytochemicals, *Pseudomonas viridiflava*

## Abstract

*Tasmannia lanceolata*, *Diploglottis bracteata* and *Syzygium aqueum* are understudied native Australian plants. This study aimed to characterise the non-anthocyanin phenolic and organic acid profiles of the aqueous extracts obtained from the leaves of *T*. *lanceolata* and fruits of *D*. *bracteata* and *S*. *aqueum* by UHPLC-Q-Orbitrap-MS/MS and UHPLC-TQ-MS/MS. A total of 39, 22, and 27 non-anthocyanin polyphenols were tentatively identified in *T*. *lanceolata*, *D*. *bracteata*, and *S*. *aqueum* extracts, respectively. Furthermore, sugars and ascorbic acid contents as well as in vitro antioxidant and antimicrobial activities of the extracts were determined. Response surface methodology was applied to achieve an extract blend with a strong inhibitory effect against *Pseudomonas viridiflava*, the main cause of soft rot in vegetables, *Bacillus subtilis*, *Rhodotorula diobovata* and *Alternaria alternata*. The identified compounds including organic acids (e.g., quinic, citric and malic acids) and polyphenols (e.g., catechin, procyanidins, and ellagitannins) might contribute to the observed antimicrobial activity. Furthermore, this study provides the most comprehensive phenolic profiles of these three underutilised native Australian plants to date.

## 1. Introduction

Exploring native plants as potential sources of bioactive compounds for a range of applications in the food industry, such as preservatives (either antioxidants or antimicrobials), flavouring agents and functional ingredients, is currently of increasing interest. Australia is the native habitat for a diverse range of plant species—over 25,000—that most of them have evolved to suit the often-harsh growing conditions and have been long used by Indigenous communities for culinary or medicinal purposes. Several studies have reported the diverse phytochemical composition and health-enhancing effects of native Australian plants [1,2]. Many of them are still largely unknown, and most of them have not yet been studied for their chemical and nutritional composition as well as biological activities.

Analysis of native Australian plants may thus offer promising prospects for finding phytochemicals with strong bioactive properties. Therefore, three underutilised plants of the native Australian flora were investigated in the present study. *Syzygium aqueum* (Burm. F.) Alston (Myrtaceae), commonly known as the watery rose-apple or lillypilly, is native to a region ranging from tropical Asia to north Queensland (Australia), and its fruits and leaves have been traditionally used as an antibiotic agent [3]. *Diploglottis bracteata* (Sapindaceae), known as boonjee tamarind, is native to the tropical rainforest regions of northeast Queensland (Australia) that produces a three-segmented orange fruit on a large bushy tree [4]. A review of the literature revealed limited or no peer-reviewed information about this species. *Tasmannia lanceolata* Poir. (Winteraceae), commonly known as Tasmanian/mountain pepper, originates from Tasmania and the southeast regions of Australia, with reported inhibitory activity against a wide spectrum of microorganisms [1,5]. It is a shrub of 2–5 m height with dark green aromatic leaves and small fleshy black berries that have a pleasant spicy flavour and sharp aroma. Different parts of the plants such as bark, leaves and berries have been historically used as herb/spice in culinary preparations and as therapeutic agents by Indigenous communities [6].

Microbial food spoilage and food-borne outbreaks are still the issues of serious concern in the food industry, which cause major economic loss and affect the company’s reputation. Synthetic chemicals as antimicrobial agents (e.g., chlorine dioxide, potassium sorbate) have been extensively utilised in food industry to tackle spoilage and pathogenic microorganisms. However, their prolonged application raised several major concerns about the potentially harmful effects of chemical residues on/in food on consumer health and the environment, as well as their high variability in efficacy due to the emergence of microbial resistance [7]. The latter is a constantly shifting challenge that is also becoming a threat to human health. The raised awareness among consumers about chemical preservatives led to an increase in demand for natural alternatives, which has become the driving force behind the scientific effort to find effective natural antimicrobials to minimise the use of chemicals. There has been strong empirical evidence for the effective antimicrobial activity of several plant extracts such as Korean mint (*Agastache rugosa*) flower, stem, and leaves [8], as well as raspberry (*Rubus idaeus*) fruit [9].

Generally, different plant structures such as fruits and leaves are rich in various phytochemicals, including polyphenols, which are products of the plant’s secondary metabolism. These specialised metabolites perform important protective functions in plants against external stressors such as invading pathogens, drought, and ultraviolet radiation [10]. These interesting features of polyphenols have resulted in them being the focus of more detailed studies during the last decades, which revealed their wide range of bioactive properties such as antimicrobial and antioxidant activities [11,12]. The presence of various bioactive components in plants and in turn plant-derived extracts represents an invaluable potential for not only nutritional but also preservative purposes. Accordingly, the application of plant extracts as natural preservatives has recently become an area of growing interest for food manufacturers, which also contributes to clean-label food products. Examples launched by several ingredient manufacturers that are presently available for the food industry include GUARDIAN^®®^ based on rosemary, green tea and acerola extracts (Danisco DuPont, Itasca, IL, USA), Berry Very^®®^ based on moso bamboo extract (Takex Labo, Osaka, Japan), NJ, USA), and XtraBlend RN^®®^ based on rosemary and spinach extracts (Naturex, Avignon, France).

The phytochemical composition, antioxidant, and antimicrobial properties of aqueous extracts of *T*. *lanceolata* leaves as well as *S*. *aqueum* and *D*. *bracteata* fruits were investigated in the present study. To the best of our knowledge, this is the first study reporting on the untargeted identification of non-anthocyanin polyphenolic compounds (by UHPLC-Q-Orbitrap-MS/MS), organic acids and sugars (by UHPC-TQ-MS/MS) in these three underutilised plants. Furthermore, extracts were screened for antimicrobial activity and antioxidant capacity, and an effective antimicrobial extract blend against common spoilage microorganisms in vegetables was optimised using an I-optimal design.

## 2. Materials and Methods

### 2.1. Chemicals

The analytical reagents and solvents (HPLC grade) used for analysis were purchased from Sigma-Aldrich (Castle Hill, NSW, Australia), Fisher Chemical (Loughborough, UK), BDH AnalaR (Kilsyth, VIC, Australia), Univar Ajax Chemicals (Sydney, NSW, Australia), and Chem-Supply (Gillman, SA, Australia). The sugar standards, including glucose, fructose, and sucrose; L-ascorbic acid; organic acids including citric acid, fumaric acid, isocitric acid, maleic acid, malic acid, quinic acid, shikimic acid, succinic acid and tartaric acid; phenolic standards, including caffeic acid, catechin, chlorogenic acid, p-coumaric acid, ellagic acid, epicatechin, gallic acid, kaempferol, luteolin, myricetin, quercetin, quercetin-3-glucoside and rutin, were purchased from Sigma-Aldrich. Microbial media were purchased from Thermo Fisher Scientific (Melbourne, VIC, Australia).

### 2.2. Plant Material and Extraction

Deseeded and frozen *S*. *aqueum* (Cape York lillypilly) and *D*. *bracteata* fruits collected in December 2017 and 2020 were purchased from Rainforest Bounty (Atherton, QLD, Australia). Air-dried *T*. *lanceolata* leaves collected in 2019 were donated by Diemen Pepper (Birchs Bay, TAS, Australia). Fruits were freeze-dried, and the dried fruits and leaves were separately ground, homogenised, and stored at −20 °C until extract preparations.

Water (Milli-Q, Millipore, Burlington, MA, USA) was brought to a boil, and then plant material was added with the ratio of 1:20 (plant:water; *w*/*v*) for *S*. *aqueum* and 1:10 for the other two. The extraction was performed under constant magnetic agitation (30 min, 70 °C), followed by centrifugation (4700 rpm, 10 min, 10 °C; Sorvall RC 12BP^+^, Thermo Scientific, Osterode am Harz, Germany). The pellet was re-extracted (2 h, 25 °C) and centrifuged. The two supernatants were mixed, vacuum-filtered through Whatman No. 1 filter paper, concentrated using a vacuum evaporator (DUC-23050-H00, miVac-Genevac, Ipswich, UK), freeze-dried (DynaVac, Lindner and May, Windsor, QLD, Australia), and kept at −20 °C until further use. The extraction yield (%) was calculated by dividing the weight of dried extract by the weight of plant material used and multiplying by 100. Thus, the extraction yields for water extracts of *T*. *lanceolata* leaves, *D*. *bracteata* fruits and *S*. *aqueum* fruits were 29.09 ± 0.41, 63.93 ± 0.83 and 56.17 ± 3.19%, respectively.

All experiments were carried out in triplicate. The details of the chromatographical separation and mass spectrometry are presented in Table 1.

### 2.3. Sugar Analysis

The extraction and analysis of soluble sugars were carried out according to the method described by Hong and colleagues [13]. External calibration curves of sugar standards (2.1–260 µg.mL^−1^) were used for quantification.

### 2.4. Vitamin C Analysis

The extraction and analysis of vitamin C (L-ascorbic acid (L-AA) and dehydroascorbic acid (DHAA)) were carried out as described by Phan and colleagues [14]. An external L-AA (1.5–76.3 mg.mL^−1^) calibration curve was used for quantification.

### 2.5. Organic Acid Analysis

Analysis of organic acids was conducted as reported by Moldoveanu and colleagues [15], with some modifications. Approximately 0.5 g of extract powder was mixed with 0.2 M HCl and vortexed for 1 min, followed by sonication (15 min, 25 °C). The mixture was then shaken by a reciprocating shaker for 1 h, followed by centrifugation (3900 rpm, 10 min). The supernatant was collected, and the pellet was re-extracted two more times as described above. The supernatants were then combined and filtered (0.22 µm, PTFE). External calibration curves using a mix of organic acid standards (0.2–1030 µg.mL^−1^) prepared in aqueous formic acid (1%; *v*/*v*) were used for quantification.

### 2.6. Total Phenolic Content (TPC)

The TPC of the extracts was determined using the Folin–Ciocalteu method as described by Singleton et al., 1999 [16]. The results were reported as mg gallic acid equivalents per g extract.

### 2.7. DPPH Radical Scavenging Capacity

The DPPH radical scavenging capacity assay was carried out according to the method described by Brand-Williams and co-workers [17] with slight modifications (equal aliquots of 0.1 mM DPPH and sample). The results were reported as IC_50_ (μg.mL^−1^).

### 2.8. HRAM Analysis and Tentative Identification of Non-Anthocyanin Phenolic Compounds

Approximately 0.5 g of extract powder was resuspended in 80% methanol containing 1% formic acid and passed through a 0.22-µm PTFE filter after centrifugation. Thirteen phenolic standards (Section 2.1) were prepared in methanol and injected into the UHPLC-MS/MS system either individually or in combination. A full MS scan in negative mode with the range of 100–1200 m/z followed by an all-ion fragmentation scan in the range of 80–1000 m/z was performed to acquire the MS and MS^2^ data. The MS characteristics of each peak detected in the UV spectra were determined based on the retention time, isotope distribution of neutral mass and the MS^2^ fragments spectra. Compound identification was carried out by manual comparison with injected standards (targeted identification) and matching with MS data reported in the literature and online database [18] to tentatively identify the unknown compounds (untargeted identification).

### 2.9. Antimicrobial Activity

#### 2.9.1. Design of Experiments

Response surface methodology using Design Expert v.11.1.2.0 (Stat-Ease Inc., Minneapolis, MN, USA) was employed to study the effect of varying extract concentrations in the blend on the inhibitory activity against the selected spoilage microorganisms and subsequently to determine the optimal extract concentrations. A 17-run Box–Behnken design consisting of five replicate centre points was developed with *T*. *lanceolata* (A, 0–10% (% is equivalent to g per 100 mL)), *D*. *bracteata* (B, 0–10%) and *S*. *aqueum* (C, 0–10%) as independent variables (Table 2). This resulted in various extract content combinations, with 25% as the highest extract content in the blend. Moreover, a 14-run randomised Simplex–Lattice mixture design with one central point was developed, and the total concentration of the extracts “%*T*. *lanceolata* (component A) + %*D*. *bracteata* (component B) + %*S*. *aqueum* (component C)” was constrained to 10% with each extract ranging from 0 to 10% (Table 2). The effect of independent variables on the studied responses was determined through the model equations and visually expressed in 3D contour plots. A polynomial equation was used to fit the experimental data and establish the relations between the independent variables and the obtained responses. The lack-of-fit test, coefficient of determination (R^2^), and adjusted R^2^ were used to assess the validity and adequacy of the fitted model. The blend of extracts was optimised by the desirability function to maximise the inhibitory activity against the studied microorganisms.

#### 2.9.2. Agar Well Diffusion Assay

*Pseudomonas viridiflava*, *Bacillus subtilis*, *Rhodotorula diobovata* and *Alternaria alternata* were taken from a culture collection of the University of Queensland (Coopers Plains, QLD, Australia), which were isolated and identified from fresh-cut capsicums (unpublished data) and stored at −80 °C. Briefly, the inoculums (10^6^ CFU.mL^−1^) of overnight-grown bacteria and yeast, and 5-day-old mould were spread on Mueller Hinton (bacteria) and potato dextrose (fungi) agar plates. Three 8 mm wells were punched in the plate and filled with 100 μL of the sample. Plates were incubated at 25 °C for 48 h (fungi) and 30 °C for 24 h (bacteria). The inhibition zone diameter (mm) was measured by a digital calliper (±0.01 mm, Craftright, China) and subtracted from the well diameter. The sensitivity according to “diameter of inhibition zone” can be categorised as follows: <8 mm not sensitive, 9–14 mm sensitive, 15–19 mm very sensitive, and >20 mm extremely sensitive [19].

### 2.10. Statistical Analysis

All measurements were performed in triplicate, and the results were expressed as the mean value ± standard deviation. A one-way ANOVA was used to analyse the results using SPSS software (version 27; IBM Institute Inc., Armonk, NY, USA). Tukey’s HSD test with a 95% confidence interval was used to compare the differences between means.

## 3. Results and Discussion

### 3.1. Chemical Composition

Table 3 presents data on soluble sugars, vitamin C and organic acids of aqueous extracts derived from *T*. *lanceolata* leaves, *D*. *bracteata* fruits and *S*. *aqueum* fruits. As expected, more sugar and vitamin C was found in the fruit extracts than in leaves. The *D*. *bracteata* extract showed the highest total content of sugars (ca. 34 g. 100 g^−1^ dw) and vitamin C (2.43 mg. 100 g^−1^ dw), followed by *S*. *aqueum* and *T*. *lanceolata*. The variation in the sugar content is associated with differences in plant species (i.e., genetic), sun exposure due to the canopy, respiration, and ripening rates of fruits, as well as the presence and activity of specific enzymes that are involved in sugar metabolism [20]. Fructose was found to be the most abundant sugar in both fruit extracts, while sucrose was the major sugar found in *T*. *lanceolata* leaves extract. The low content of sucrose in fruit extracts can be attributed to the ripening phenomenon that causes sucrose conversion to fructose and glucose [21].

To the best of our knowledge, the organic acid profile was reported for the first time for the studied extracts. Roughly seven organic acids were identified in the extracts that exhibited very different profiles (Table 3). Quinic and citric acids were the most abundant (39.38 and 38.59% of total acids, respectively) in *S*. *aqueum* extract, while malic and shikimic acids were the most abundant (84.28% and 48.05% of total acids, respectively) in *D*. *bracteata* and *T*. *lanceolata* extracts, respectively. A considerably higher total content of organic acids was detected in *S*. *aqueum* extract (74.64 g. 100 g^−1^ dw) compared to 26.33 g. 100 g^−1^ dw in *D*. *bracteata* and 11.03 g. 100 g^−1^ dw in *T*. *lanceolata* extracts. The concentration of organic acids in fruits and leaves depends on sugar concentrations and their use for respiration. Several studies have shown the beneficial effects of organic acids not only as antibacterial agents but also on human health, including their involvement in iron absorption, reduction of levels of circulating glucose and cholesterol, and anxiolytic effects [22,23]. Quinic acid, for example, has exhibited anti-neuroinflammatory and radioprotection effects [24], as well as anti-HIV-1 activity [25].

Our results demonstrated that the antioxidant capacity of the extracts was directly related to the total phenolic content. The TPC value of 123.47 mg GAE.g^−1^ dw was found in *T*. *lanceolata* leaves extract, which showed a strong antioxidant capacity (DPPH IC_50_ value of 36.59 µg.mL^−1^). These results were in good agreement with those reported by Alderees and colleagues, who found 157.4 mg GAE.g^−1^ dw in an aqueous extract of Tasmanian pepper leaves [26]. On the other hand, the fruit extracts with low contents of TPC had a considerably low antioxidant capacity (Table 3). Unlike the fruits’ extracts, leaves extracts with strong antioxidant activity can be used to reduce oxidative stress and contribute to preventing damage by reactive species. Several studies have also shown low values of TPC in *S*. *australe* (2.14 mg GAE.g^−1^ dw) and *S*. *luehmannii* (2.23 mg GAE.g^−1^ dw) [27,28]. The accumulation of phenolic compounds in different plant tissues is influenced by environmental conditions such as temperature, sun exposure and other weather conditions, which may explain the observed differences in the studied extracts. For instance, the observed higher TPC in Tasmanian pepper leaves could be attributed to the increased expression of genes associated with flavonoid biosynthesis due to high sun exposure [29]. Generally, the biosynthesis of phenolic compounds in plants is the result of a collection of regulatory signals, including developmental (e.g., during anthocyanin production during fruit and flower development) and environmental (e.g., protection against abiotic and biotic stresses) signals [30].

### 3.2. Identification of Non-Anthocyanin Polyphenols

Table 4, Table 5 and Table 6 present the data on untargeted screening and tentative identification of non-anthocyanin polyphenols in the aqueous extracts of *T*. *lanceolata* leaves, *D*. *bracteata* fruits and *S*. *aqueum* fruits, using HRAM-UHPLC-Q/Orbitrap-MS/MS. The retention time and MS/MS fragmentation pattern were compared with the reported data in previous studies. A total of 39, 22, and 27 non-anthocyanin polyphenols were tentatively identified in *T*. *lanceolata*, *D*. *bracteata*, and *S*. *aqueum* aqueous extracts, respectively. The UHPLC-UV chromatograms, the mass spectra data of not-yet identified compounds (due to the unavailability of commercial standards and limited MS data in the literature), commercial standards used in this study, as well as representative full-scan and product ion mass spectra, are summarised in the Supplementary Materials (Figures S1–S7, Tables S1–S4).

#### 3.2.1. Phenolic Acids

Hydroxycinnamic acids were the most abundant phenolic acids detected in the *T*. *lanceolata* extract. Compounds **1**, **13**, and **14** were tentatively identified as chlorogenic acid derivatives, including hydroxydihydrocaffeoylquinic acid [31], chlorogenic acid dimer, and its isomer [32]. Chlorogenic acid (compound **12**) was identified and confirmed by a commercial standard, exhibiting m/z 191.0557 [quinic acid–H]^−^ and m/z 85.0284 as major fragment ions. Compound **8** was tentatively identified as a caffeoylquinic acid glucoside derivative, which showed product ions at m/z 515.1109 [caffeoylquinic acid glucoside–H]^−^ as well as at m/z 191.0559 [quinic acid–H]^−^ and m/z 323.0540 [caffeoyl glucosyl–H_2_O–H]^−^ that are correspondent to caffeoylquinic acid glucoside fragmentation [31]. Two coumaric acid derivatives were tentatively identified as compounds **6** and **16** with a diagnostic fragment ion at m/z 163.0394 [p-coumaric acid–H]^−^ [33]; however, compound **16** was characterised as 4-O-p-coumaroylquinic acid due to m/z 119.0495 [p-coumaric acid–H–CO_2_]^−^ as a further main fragment [34].

Hydroxycinnamic acid compounds were also tentatively identified in the *D*. *bracteata* extract. Compound **14** was tentatively assigned as a coumaric acid derivative, producing the main fragment at m/z 119.0496 [p-coumaric acid–H–CO_2_]^−^ [34]. Compound **17** was tentatively suggested as a caffeoyl glucose derivative that showed a product ion at m/z 341.607, most likely [caffeoyl glucose–H]^−^, and another at m/z 161.0609, which was reported as a caffeoyl glucose fragment [35]. Furthermore, compounds **16**, **20**, and **22** were tentatively assigned as cinnamic acid derivatives according to the main fragments produced at m/z 147.0445 [cinnamic acid–H]^−^ [35], m/z 245.1545 [heptyl cinnamate–H]^−^ and m/z 231.0989 [tetrahydrofurfuryl cinnamate–H]^−^ [18].

Three tentatively identified hydroxybenzoic acid compounds were also found in the *T*. *lanceolata* extract. Compounds **3** and **4** were tentatively assigned as protocatechuic acid-O-hexoside [36] and protocatechuic acid [37], respectively. Compound **10** may be a hydroxybenzoic acid derivative with the precursor ion at m/z 447.1867 [M–H]^−^ that dissociated to m/z 137.0238, which corresponds to a [hydroxybenzoic acid–H]^−^ adduct. One dihydroxybenzoic acid was tentatively identified in the *D*. *bracteata* extract as hypogallic acid (compound **4**), producing m/z 108.0213 [M–H–COOH]^−^ as the main fragment [38]. Interestingly, the only phenolic acids found in the *S*. *aqueum* extract were compounds **3**, **5** and **8**, belonging to the benzoic acid group, and were identified as gallic acid (confirmed by commercial standard), bergenin [39] and theacitrin A (ester derivative) [40].

#### 3.2.2. Flavonoids

Flavonoids were the most abundant compounds found in the three studied extracts (Table 4, Table 5 and Table 6). Six flavones were tentatively identified in the *T*. *lanceolata* extract. Compounds **26** and **27** with the main product ion at m/z 284.0324 [luteolin–H]^−^ were tentatively assigned as luteolin derivatives. Compound **33** produced m/z 343.1535 [M–hexose–H]^−^ and m/z 328.1302 [M–hexose–CH_3_–H]^−^ as the prominent fragments due to the loss of 162 Da hexose and a further 15 Da methyl group, and therefore was tentatively identified as luteolin-trimethyl ester-O-hexoside [41]. A potential luteolin derivative (compound **15**) was also found in the *D*. *bracteata* extract, having the characteristic fragment ions at m/z 285.0397 as a luteolin adduct and m/z 107.0130 as a luteolin derivative.

Moreover, three O-methylated flavones were detected in the *T*. *lanceolata* extract. Compound **38** was tentatively identified as diosmetin (syn: 4-O-methylluteolin) that fragmented into luteolin at m/z 284.0321 [M–CH_3_–H]^−^, and the major luteolin fragments at m/z 133.0289 and m/z 107.0131 [42]. Compounds **35** and **36** with the two characteristic fragment ions at m/z 299.0552 [4-O-methylluteolin–H]^−^ and m/z 284.0319 [luteolin–H]^−^ were tentatively assigned as diosmetin derivatives. Furthermore, compound **22** in the *D*. *bracteata* extract was tentatively identified as a wogonin derivative (an O-methylated flavone), producing a fragment ion at m/z 283.1545 [43], most likely caused by a hexose loss.

Apigenin (compound **37**) was tentatively identified as another flavone present in the *T*. *lanceolata* extract [42]. Compound **32** was tentatively assigned as an apigenin derivative due to the produced characteristic fragment at m/z 269.0450 [apigenin–H]^−^. Compound **39** fragmented into m/z 268.0371 [M–CH_3_–H]^−^ as well as the characteristic apigenin fragments at m/z 117.0337 and m/z 151.0030, and was therefore tentatively identified as apigenin-7,4′-dimethyl ether [44].

The flavonol, quercetin, was identified in the *T*. *lanceolata* (compound **34**) and *S*. *aqueum* (compound **27**) extracts, which was confirmed by a commercial standard. Two more flavonols were identified in the *S*. *aqueum* extract. Compound **26** was confirmed as myricetin using a commercial standard, and compound **7** was tentatively identified as a kaempferol derivative, with the main fragment ion at m/z 284.0318 [kaempferol–H]^−^. Catechin (compound **8**) and epicatechin (compound **12**), two flavanols, were identified in the *D*. *bracteata* extract and confirmed by commercial standards. Furthermore, compound **11** in the *S*. *aqueum* extract was tentatively assigned as gallocatechin gallate, producing the two characteristic fragment ions at m/z 125.0238 and m/z 169.0137. Compound **11** in the *D*. *bracteata* extract was tentatively identified as an eriodictyol derivative, a flavanone, with fragment ions at m/z 287.0552 as a deprotonated eriodictyol adduct and m/z 125.0239 as an eriodictyol fragment [45].

#### 3.2.3. Flavonoid Glycosides

Flavonoid glycosides were also detected in the *T*. *lanceolata* extract. Compound **17** was tentatively identified as catechin rhamnoside, showing the main fragment ion at m/z 289.0709 by a 146 Da rhamnoside residue loss. Compounds **21** and **22** were identified as rutin (syn: quercetin-3-rutinoside) and quercetin-3-O-glucoside, respectively, and confirmed by commercial standards. The characteristic fragment ions at m/z 300.0275 and m/z 300.0266 were produced by a 308 Da rutinose and 162 Da glucose loss, respectively. Compounds **24** and **23** were tentatively identified as vitexin/isovitexin and vitexin/isovitexin dimer, respectively. The MS^2^ spectra showed the characteristic fragment ions at m/z 283.0606 [M–148–H]^−^ and m/z 311.0549 [M–120–H]^−^ [34], with the latter most likely produced by a neutral loss of a glucosyl residue. Compound **23** showed a further fragment ion at m/z 431.0974, corresponding to [M–vitexin/isovitexin–H]^−^. Vitexin/isovitexin was also tentatively identified in the *S*. *aqueum* extract (compound **18**). Compounds **25** and **28** were tentatively identified as glycosylated kaempferol, showing m/z 285.0392 and m/z 285.0397 as the main fragment ions that are typical of the kaempferol aglycone [32,46], and produced by a neutral loss of 308 Da (probably rhamnoglucose) and 278 Da (probably rhamnoxylose), respectively. Furthermore, compound **30** was tentatively identified as luteolin glycoside, showing luteolin aglycone as the main fragment ion at m/z 285.0402 [M–162–H]^−^ through the neutral loss of a hexose residue such as glucose or galactose [47]; however, glucose is more likely since it is the most common hexose in nature. Compound **31** was tentatively assigned as apigenin dihexoside [48], which produced apigenin as the main fragment (m/z 269.0451) through the neutral loss of two hexosyl groups.

Two flavonoid glycosides were also detected in the *D*. *bracteata* extract. Compound **18** was tentatively identified as isorhamnetin glycoside (a flavonol glycoside) by producing the two characteristic fragment ions at m/z 299.0194 and m/z 314.0470 [49], with the latter one resulting from a hexose loss. Compound **9** was tentatively identified as a catechin glycoside by producing m/z 289.0712 (catechin) as the main fragment ion through a 158-Da loss.

A total of 11 flavonoid glycosides were found in the *S*. *aqueum* extract (Table 6). Compound **6** was tentatively identified as luteolin-3-glucoside, showing m/z 285.0385 as the main fragment ion [50] through a 162-Da loss (glucoside moiety). Compounds **15** and 14 were tentatively identified as myricetin-3-O-β-D-galactopyranoside and its isomer producing major fragment ions at m/z 316.0220 (myricetin aglycone) after a 162-Da loss (galactose) and m/z 271.0250, which corresponds to 3-O-monoglycosides [51]. Compounds **17** and **22** were tentatively assigned as dihydrokaempferol-hexoside [52] and myricetin glycoside [53] due to the neutral hexose loss and the formation of the characteristic fragment ions at m/z 287.0185 [dihydrokaempferol–H]^−^ and m/z 317.0288 [myricetin–H]^−^, respectively. Compound **20** was tentatively identified as phloretin-diglucoside, a dihydrochalcone, producing the characteristic main fragment ions at m/z 357.0947 [M–H–(2 × 120)]^−^, m/z 387.1089 [M–H–120–90]^−^, and m/z 417.1104 [M–H–(2 × 90)]^−^ [54]. Compound **21** was identified as quercetin-3-O-glucoside and confirmed by a commercial standard. Compounds **23** and **25** were tentatively identified as quercetin glycosides, producing main fragments at m/z 300.0269 and m/z 301.0339 through a 132 Da pentose loss, and at m/z 271.0248 through a 162 Da hexose loss [55]. Compound **24** was identified as phloridzin, showing the characteristic fragment ions at m/z 167.0340 and m/z 273.0750 [39], with the latter resulting from a 162 Da loss.

#### 3.2.4. Polyflavonoids

Procyanidins, also known as condensed tannins, are classified as polyflavonoids that were found in both *T*. *lanceolata* and *D*. *bracteata* extracts. Compounds **9** and **19** in the *T*. *lanceolata* extract as well as compounds **6**, **7** and **10** in the *D*. *bracteata* extract were tentatively identified as B-type procyanidin dimers (or (epi)catechin-(epi)catechin) with a precursor ion at m/z 577.1331. The characteristic fragment ions included m/z 289.0710 [M–H–288]^−^ through interflavonoid C–C linkage cleavage, m/z 125.0237 [M–H–288–164]^−^ through heterocyclic ring fission (HRF) of the C-ring of the dimer, m/z 407.0778 [M–H–152–18]^−^ through Retro–Diels–Alder (RDA) fission of the heterocyclic ring followed by a water loss [56], and m/z 245.0814 [M–H–288–44]^−^ [32,47]. However, the difference in their fragmentation patterns can be attributed to the differences in monomeric flavan-3-ol unit linkages, leading to different isomers’ formation [57]. Compounds **18** and **20** in the *T*. *lanceolata* extract were tentatively assigned as procyanidin dimer monoglycoside, producing the diagnostic fragment ions at m/z 289.0714 (probably formed through quinone methide cleavage [58]), m/z 245.0814, m/z 587.1086 (152 Da loss through RDA fission) and m/z 569.0995 (152-Da loss with a further 18 Da loss through dehydration) [56]. Compound **2** in the *T*. *lanceolata* extract [59] and compound **13** in the *D*. *bracteata* extract [50] were tentatively identified as procyanidin trimers, showing the characteristic procyanidin fragmentation pathway. Furthermore, compound **5** (m/z 593.1262) in the *D*. *bracteata* extract was tentatively assigned as prodelphinidin A-type [50].

#### 3.2.5. Tannins

Tannins, including one complex and seven hydrolysable tannins, were only detected in the *S*. *aqueum* extract. Compound **12** was tentatively identified as theasinesin A, a complex tannin, with the main fragment ions at m/z 741.0924 [M–H–152–18]^−^ and m/z 571.0792 [M–H–152–18–170]^−^ [39]. Compound **19** was identified as ellagic acid (m/z 300.9978) and confirmed by a commercial standard. This was detected as one of the characteristic fragment ions of five tentatively identified ellagitannins found in the *S*. *aqueum* extract. Compounds **4**, **10** and **13** were tentatively assigned as castalagin [60], chebulagic acid [61] and casuarinin [62]. Compound **16** was tentatively identified as an ellagic acid derivative owing to the diagnostic fragment ions at m/z 299.9907 and m/z 300.9964. The presence of fragment ions at m/z 169 and m/z 301 reveals that the hydrolysable tannin molecule contains a simple galloyl ester and a hexahydroxy-diphenoyl (HHDP) moiety [63]. The observed fragmentation pattern of hydrolysable tannins was in agreement with those found in the literature [64], showing the characteristic loss of galloyl, HHDP, HHDP-glucose, and galloyl-HHDP-glucose. Compound **9** was tentatively identified as a galloylated tannin compound showing fragment ions at m/z 125.0237, m/z 169.0139 and m/z 633.0800.

#### 3.2.6. Other Polyphenols

Other tentatively identified polyphenols detected in the *T*. *lanceolata* extract were compound **5** as a hydroxybenzaldehyde derivative [35], two stilbenes, including compound **7** as a piceatannol derivative [65] and compound **11** as pelargonidin-3-pentoside [66], producing the main fragment ions at m/z 121.0289 [hydroxybenzaldehyde acid–H]^−^, m/z 243.0567 [piceatannol–H]^−^ and m/z 271.0964 [pelargonidin–H]^−^. Compound **29** was tentatively assigned as cinochonain l (m/z 451.1031), an alkaloid, showing the diagnostic fragment ions previously reported [36]. A coumarin derivative (compound **3**) with the main fragment at m/z 147.0441 corresponding to coumaric acid was tentatively identified in the *D*. *bracteata* extract. Furthermore, compound **20** was tentatively assigned as a carnosic acid derivative, as the fragment ions at m/z 331.1887 and m/z 269.0455 correspond to carnosic acid and its fragment adducts. Moreover, organic acids, including malic acid [47] and citric acid [67], were tentatively identified in the *D*. *bracteata* (compound **1**) and *S*. *aqueum* (compounds **1** and **2**) extracts.

### 3.3. Antimicrobial Activity of Extract Blends

The aqueous extracts of plant tissues are rich in various phytochemicals that are readily soluble in water and influence their antimicrobial activity. However, the inhibitory activity can be improved by mixing two or more plant extracts through the synergistic interactions between their major and minor constituents. Hence, RSM optimization through Box–Behnken and Simplex–Lattice designs was performed for the first time on *T*. *lanceolata*, *D*. *bracteata*, and *S*. *aqueum* aqueous extracts as potential natural preservatives to find two optimised blends exhibiting the highest inhibitory activity against the growth of common spoilage microorganisms in vegetables. Table 2 shows the experimental matrix designs and results using Box–Behnken and Simplex–Lattice. Quadratic polynomial equations for predicting the inhibitory activity of extract blends against *P*. *viridiflava*, *B*. *subtilis*, *R*. *diobovata* and *A*. *alternata* were determined by multiple regression analysis of the experimental data obtained from Box–Behnken (Equations (1)–(4)) and Simplex–Lattice (Equations (5)–(8)) designs. In order to fit the data to the respective models, Box–Cox transformation and stepwise model reduction were performed, if needed. The resulting equations, including the hierarchy required for insignificant (*p* > 0.05) and significant (*p* < 0.05) terms, are given below:

(R_1_)^1.35^ = 46.59−0.07A + 10.30B + 18.16C−7.42BC−3.87B^2^−7.02C^2^ (1)

R_2_ = 15.22 + 3.66A + 1.53B + 3.94C−1.04AB−1.65AC−1.99BC−3.31A^2^−0.58B^2^−1.78C^2^ (2)

R_3_ = 20.22 + 11.51A + 0.89B + 0.49C−2.82BC−6.53A^2^−2.00B^2^−2.39C^2^ (3)

R_4_ = 2.74 + 2.17A−0.01B + 0.69C−1.50BC + 0.87A^2^−1.09B^2^ (4)

R_5_ = 0.32A + 13.39B + 18.69C + 11.74AB + 20.46AC−0.91BC (5)

R_6_ = 9.45A + 7.28B + 11.80C + 12.24AB + 13.38AC (6)

(7) $$\sqrt{R_{7}\;+\;0.5}=4.54A+0.72B+0.71C+7.20AB+8.25AC$$

(8) $$\sqrt{R_{8}\;+\;0.5}=2.38A+0.67B+1.05C+3.28AB+2.11AC$$

Table 7 summarises the statistical parameters obtained by performing ANOVA to check the reliability and adequacy of the developed models (details are given in Supplementary Materials, Tables S5–S12). The R^2^ values were in the range of 0.89–0.99 for the Box–Behnken design and 0.77–0.99 for the Simplex–Lattice design, showing sufficient model accuracy. This indicates that not only can the fitted models explain (*p* < 0.05) most of the variability in the experimental data, but there is also a strong correlation between the experimental and predicted values. In addition, the low reliability of the developed models for *A*. *alternata* in the Box–Behnken design and for *B*. *subtilis* and *A*. *alternata* in the Simplex–Lattice design was indicated by low R^2^ values, although they can be used to generate adequate desirability models. The insignificant *p*-values (*p* > 0.05) of the lack-of-fit test indicated that the models fit the inhibitory activity of extract blends and confirmed the reliability of the predicted models. However, the significant *p*-values (*p* < 0.05) of the lack-of-fit test for the inhibitory activity against *R*. *diobovata* demonstrated that the obtained quadratic models did not fit well in these experimental designs, even after Box–Cox transformation and stepwise model reduction, and therefore, these models cannot be used for predictions.

**Figure 1:**
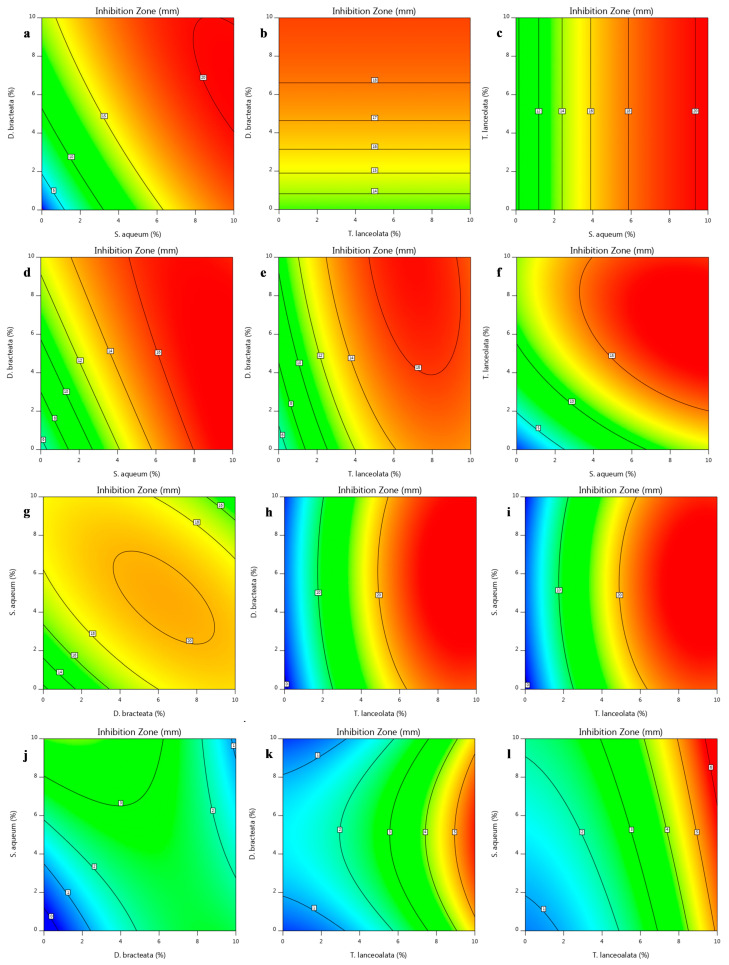
Contour plots for the inhibition zone of extract blends against *Pseudomonas viridiflava* (**a**–**c**), *Bacillus subtilis* (**d**–**f**), *Rhodotorula diobovata* (**g**–**i**) and *Alternaria alternata* (**j**–**l**) as a function of independent factors (Box–Behnken design).
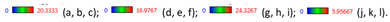

**Figure 2:**
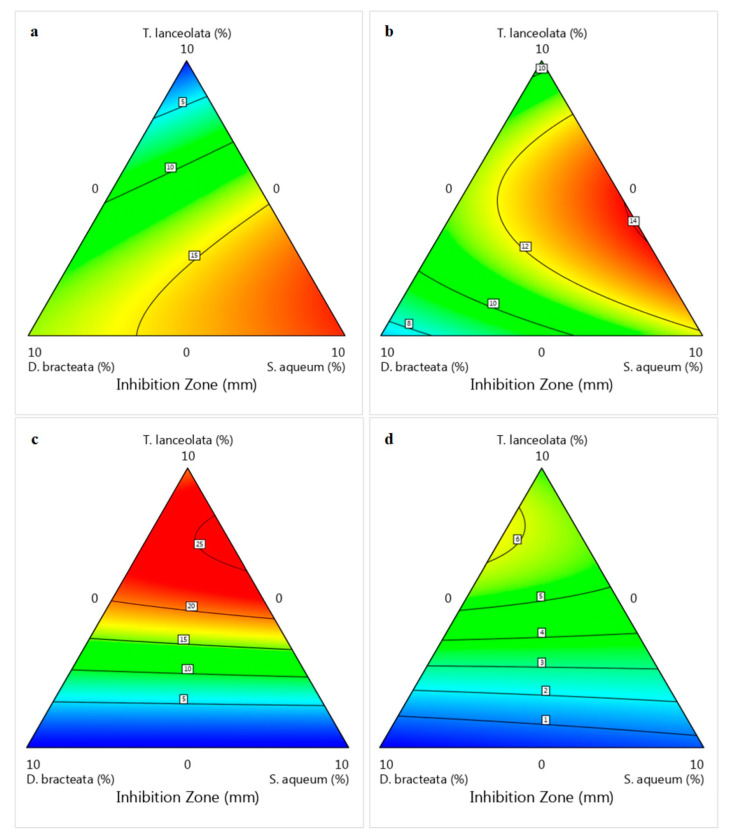
Contour plots for the inhibition zone of extract blends against *Pseudomonas viridiflava* ((**a**); 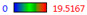), *Bacillus subtilis* ((**b**); 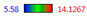), *Rhodotorula diobovata* ((**c**); 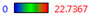) and *Alternaria alternata* ((**d**); 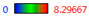) as a function of independent factors (Simplex–Lattice design).

The growth inhibitory zone of Gram-negative *P*. *viridiflava*, the major cause of soft rot in vegetables such as capsicum, was in the range of 0–20.33 mm (Box–Behnken) and 0–19.52 mm (Simplex–Lattice) (Table 2). The most potent antibacterial extract was *S*. *aqueum*, followed by *D*. *bracteate*. In the presence of 5% *S*. *aqueum* and *D*. *bracteata* extracts, inhibition zones of 13.20 and 9.02 mm were observed, respectively, while *T*. *lanceolata* extract did not exhibit any inhibitory effect against *P*. *viridiflava* (Table 2). Increasing the concentration of extracts to 10% gave rise to an increase in the inhibitory activity against *P*. *viridiflava* by 2 and 1.5 times (*S*. *aqueum* and *D*. *bracteata*, respectively), but did not improve *T*. *lanceolata* activity. Blending the *S*. *aqueum* extract with the other two did not improve its ability to inhibit *P*. *viridiflava* growth, although it assisted in improving the antifungal activity. However, lower inhibition of *P*. *viridiflava* was observed using blends containing <5% *S*. *aqueum* (Simplex–Lattice). The linear terms in both designs were shown to affect (*p* < 0.05) the bacterial inhibitory activity, except for *T*. *lanceolata* extract, which had an insignificant (*p* > 0.05) inhibitory influence on *P*. *viridiflava* growth (Box–Behnken design). The interactions and quadratic terms of aqueous extracts indicated an inverse relationship with the bacterial inhibitory activity of the blend according to the Box–Behnken design (Equations (2) and (3)). However, the interaction terms in the Simplex–Lattice design led to a significant (*p* < 0.05) increase in antibacterial activity (Equations (6) and (7)). This was further illustrated in two-dimensional contour plots developed from the fitted model equations (Figure 1a–f). Both designs demonstrated the greatest impact of *T*. *lanceolata* content on the fungal inhibitory activity, with yeast being more sensitive than mould (Figure 1g–l and Figure 2c,d). No antifungal activity was observed by *S*. *aqueum* and *D*. *bracteata* extracts alone, whereas 5% *T*. *lanceolata* extract showed an inhibitory zone of 9.17 mm against *R*. *diobovata* which was doubled by increasing the concentration to 10%, and a 5.34 mm inhibitory zone was observed against *A*. *alternata*. The highest inhibitory zones against *A. alternata* were 8.29 and 5.96 mm using experimental runs 3 (Simplex–Lattice) and 4 (Box–Behnken), respectively, with different extracts’ combinations. This indicates the potential of the Simplex–Lattice mixture design, unlike Box–Behnken, to unfold the synergistic effect of the extracts on the blend’s antifungal activity at the ratio of 2/3 *T*. *lanceolata*, 1/6 *D*. *bracteata*, and 1/6 *S*. *aqueum*. This was also confirmed by ANOVA and the interaction terms of the developed models.

The observed antimicrobial activity is mainly attributed to the synergistic effect of organic acids and phenolic compounds, which has been well demonstrated [68] and is considered an added benefit of using fruit extracts as preservatives. Partially hydrophobic biphenols can bind with the microbial outer membrane and cause structural changes leading to enhanced membrane permeability, leakage of vital intracellular constituents, and disruption of metabolism [69]. The antimicrobial properties of different phenolic compounds have been extensively studied, such as catechin [70], gallic acid [71], chlorogenic acid [72], and hydrolysable tannins [73], which were identified as major phenolic compounds in the studied extracts. Procyanidin, a tentative major phenolic compound in *D*. *bracteata* and *T*. *lanceolata* extracts, has been shown to affect the strength of the lipopolysaccharide outer barrier in Gram-negative bacteria as observed by cranberry polyphenols [74]. Moreover, phenolic compounds with antioxidant activity can bind the essential growth nutrient “iron” and therefore inhibit microbial growth. Guo and colleagues observed a considerable iron binding capacity by quercetin in low pH environments [75], which is one of the major phenolics in the studied extracts, including *S*. *aqueum,* which also contains high amounts of organic acids such as quinic acid. The presence of organic acids, on the other hand, enhances the bacteria’s susceptibility to phenolic sublethal damage by reducing pH in the extra/intracellular environment, causing chemical gradient collapse, and interrupting metabolic pathways [76]. In addition, several studies have reported the wide-spectrum antibacterial activity of quinic acid, citric acid, and malic acid, and their combinations, which were identified as major organic acids in *S*. *aqueum* and *D*. *bracteata* extracts (Table 3). The presence of sugars in the extracts can also contribute to the observed antimicrobial activity, as was suggested by Lacombe and co-authors. The authors reported the effect of sugar fractions of cranberry juice on its antimicrobial activity [74], which can be attributed to the osmotic effect of sugar compounds on microbial cells. However, this needs to be further investigated. The lower antimicrobial activity against *A*. *alternata* can be attributed to the mould’s phenylacrylic acid decarboxylase system for degrading high concentrations of organic acids for their spores to survive and outgrow [76]. However, the presence of chlorogenic acid, a major compound in *T*. *lanceolata* extract, can contribute to its antifungal activity. Several studies have shown the fungicidal activity of chlorogenic acid and its derivatives, which occurs through fungal cell lysis and permeabilization of the spore membrane [77]. Nevertheless, further studies are needed to understand the antimicrobial mechanism of these extracts and the role of sugars, organic acids, and phenolic compounds, as well as major and minor compounds.

The RSM desirability function was used to optimise the blends and maximise the antimicrobial activity. The two extract blends containing “9.35% *T*. *lanceolata*, 5.00% *D*. *bracteata* and 5.00% *S*. *aqueum*” and “4.72% *T*. *lanceolata* and 5.28% *S*. *aqueum*”, respectively, presented the best combinations based on the models developed through Box–Behnken and Simplex–Lattice designs. The d-values for the optimised combinations were 0.927 and 0.822 (Box–Behnken and Simplex–Lattice, respectively), indicating that about 93% and 82% of desirability in statistical optimisation were satisfied. The RSM models were tested by performing an external validation using the optimised extract blends (Table 8). The experimental values for inhibitory activity were within the ±95% prediction limits proposed by the regression models, which confirms the reliability and predictivity of the developed models.

## 4. Conclusions

To the best of our knowledge, this study provides, for the first time, information about organic acids and non-anthocyanin polyphenols in the aqueous extracts from *T*. *lanceolata* leaves, *D*. *bracteata* and *S*. *aqueum* fruits. The potential of aqueous extracts containing various phytochemicals such as organic acids and non-anthocyanin polyphenols to inhibit the growth of spoilage microorganisms, in particular *P*. *viridiflava,* which causes soft rot in a wide range of vegetables, was also demonstrated. The results obtained in this study could suggest various value-added applications for these plant materials and their extracts. Indeed, being a high source of bioactive compounds with antioxidant and antimicrobial properties such as polyphenols, these plants could be valorised as an industrial source of bioactive compounds, which will find application as effective alternatives to conventional chemical preservatives in the food, pharmaceutical, and cosmetic sectors. However, further studies are needed to confirm the identity of the tentatively identified compounds, to assess the impact of harvest time and storage conditions on the polyphenol/phytochemical composition, and to find stronger antifungal plant extracts.

## Figures and Tables

**Table 1:** Details of chromatographical and mass spectrometry analysis of soluble sugars, vitamin C, organic acids, and non-anthocyanin phenolics.

Analysis	Analytical Instrument	Electrospray Ionization	Multiple Reaction Monitoring (MRM) Transition *	Stationary Phase	Mobile Phase	**Gradient Program**	**Reference**
Sugars	Shimadzu Nexara X2 UHPLC, (Kyoto, Japan) equipped with a triple quadrupole mass spectrometer (MS-8045, Shimadzu). Data collection and processing were performed by Lab Solutions Insight LC-MC software (v.3.2, Shimadzu).	Negative mode. Nebulizer gas flow 3 L.min^−1^, drying gas flow of 10 L.min^−1^, desolvation line temperature of 250 °C, heat block temperature of 400 °C.	Fructose (179.2 → 113.1/89.0), Glucose (179.2 → 113.1/89.0), Sucrose (341.2 → 179.2/161.2/119.1)	Acquity UPLC BEH Amide column (100 × 2.1 mm i.d.; 1.7 µm particle size; Waters, Dublin, Ireland) at 40 °C.	Mobile phase A: 80% aqueous acetonitrile containing 0.1% NH_4_OH. Mobile phase B: 0.1% aqueous NH_4_OH.	0% B, 1 min; linear increase to 40% B, 7 min; conditioning, 1 min; re-equilibration, 3 min. Flow rate of 0.2 mL.min^−1^.	[13]
Vitamin C	Waters UPLC-PDA system. Detection was carried out at 245 nm.	-	-	Waters HSS-T3 column (100 × 2.1 mm i.d.; 1.8 µm particle size) at 25 °C.	Mobile phase: 0.1% aqueous formic acid.	Flow rate of 0.3 mL.min^−1^.	[14]
Organic Acids	Shimadzu Nexera X2 UHPLC system, equipped with a MS-8045-TQ-mass spectrometer (0.2 µL injection). Data collection and processing were performed using Lab Solutions Insight LC-MS software (v.3.2, Shimadzu).	Negative mode. Nebulizer gas flow 3 L.min^−1^, drying gas flow of 10 L.min^−1^, desolvation line temperature of 250 °C, heat block temperature of 400 °C.	Succinic acid (117.00 → 73.00/99.10), Malic acid (133.00 → 114.95/70.95), Tartaric acid (149.00 → 87.00/72.85), Fumaric acid (115.00 → 71.05/26.95), Maleic acid (115.00 → 70.95/27.00), Isocitric acid (191.00 → 110.95/173.00), Citric acid (191.00 → 110.95/86.90), Shikimic acid (173.15 → 92.90/111.00), Quinic acid (191.00 → 84.95/92.90).	Waters HSS-T3 column (150 × 2.1 mm i.d.; 1.8 µm particle size) maintained at 40 °C.	Mobile phase A: 0.1% aqueous formic acid. Mobile phase B: 0.1% methanolic formic acid.	0% B, 1 min; 5% B, 7 min; 50% B, 8 min; 50% B, 9 min; 0% B, 9.10 min; 0% B, 12 min. Flow rate of 0.2 mL.min^−1^.	[15]
Non-anthocyanin Phenolic Compounds	HRAM Thermo Q Exactive Orbitrap spectrometer (Thermo Fisher Scientific, Melbourne, VIC, Australia) equipped with an Ultimate Dionex 3000 RS UHPLC-UV/Vis detector (Thermo Fisher Scientific). UHPLC-UV chromatograms were recorded at 280 and 360 nm. Data processing was performed with Xcalibur software (v.4.1, Thermo).	Negative mode. Collision energy of 25 V, resolving power of 70,000 FWHM, 3 × 10^6^ AGC (automatic gain control) target, injection time of 200 ms (for peak identification).	-	Waters HSS-T3 column (150 × 2.1 mm i.d.; 1.8 µm) at 40 °C.	Mobile phase A: 0.1% aqueous formic acid, Mobile phase B: 0.1% formic acid in acetonitrile.	5% B, 1 min; 20% B, 5 min; 20% B, 7 min; 45% B, 12 min; 100% B, 19 min; 100% B, 22 min; 5% B, 22.1 min; 5% B, 26 min. Flow rate of 0.3 mL.min^−1^.	-

UHPLC, ultra-high-performance liquid chromatography; HRAM, high-resolution accurate mass. * MRM scan mode with optimized collision energy was employed for the targeted analysis and quantification of sugars and organic acids. Two multiple reaction monitoring transitions were used to quantify each sugar/acid and to confirm their identities according to their specific mass fragmentation pattern ([M-H]^−^ → quantifier/qualifier (m/z)).

**Table 2:** Experimental matrix for Box–Be hnken and Simplex–Lattice mixture designs and mean values of responses (*n* = 3) for inhibition zone (mm) of extract blends against the studied microorganisms.

Run	A/Component1 (TL, %)	B/Component2 (DB, %)	C/Component3 (SA, %)	*Pseudomonas viridiflava*	*Bacillus subtilis*	*Rhodotorula diobovata*	*Alternaria alternata*
**Box–Behnken**							
**1**	0	10	5	19.06	10.51	0.00	0.00
**2**	0	0	5	13.20	5.63	0.00	0.00
**3**	5	10	0	14.37	13.05	19.29	2.34
**4**	10	10	5	18.40	14.96	22.48	5.96
**5**	0	5	10	20.33	12.14	0.00	3.57
**6**	10	5	10	20.13	16.98	21.39	5.46
**7**	5	5	5	17.75	15.25	19.56	3.52
**8**	5	0	0	0.00	5.76	9.17	0.00
**9**	5	5	5	17.25	15.69	19.68	2.78
**10**	5	0	10	18.19	16.66	18.02	3.65
**11**	10	0	5	13.29	14.24	24.33	4.73
**12**	0	5	0	9.02	0.00	0.00	0.00
**13**	10	5	0	9.84	11.42	23.86	4.82
**14**	5	5	5	17.16	15.89	20.78	2.47
**15**	5	5	5	17.08	15.07	20.82	3.04
**16**	5	5	5	17.08	14.20	20.29	2.51
**17**	5	10	10	20.00	15.99	16.88	0.00
**Simplex–Lattice Mixture**							
**1**	5.00	5.00	0.00	10.05	10.04	19.05	3.71
**2**	0.00	0.00	10.00	19.52	10.87	0.00	0.00
**3**	6.67	1.67	1.67	11.14	12.37	22.74	8.29
**4**	5.00	5.00	0.00	9.15	12.29	18.42	5.24
**5**	1.67	6.67	1.67	13.58	12.55	5.18	1.76
**6**	1.67	1.67	6.67	16.48	12.86	5.05	1.69
**7**	0.00	10.00	0.00	14.17	7.58	0.00	0.00
**8**	0.00	5.00	5.00	16.36	10.42	0.00	0.00
**9**	3.33	3.33	3.33	13.60	10.99	16.29	3.28
**10**	10.00	0.00	0.00	0.00	9.26	19.50	4.47
**11**	10.00	0.00	0.00	0.00	9.80	21.26	5.34
**12**	0.00	0.00	10.00	18.15	12.00	0.00	1.76
**13**	5.00	0.00	5.00	14.39	14.13	20.93	3.67
**14**	0.00	10.00	0.00	12.75	5.58	0.00	0.00
**Positive/Negative Controls**							
Streptomycin (20 µg.mL^−1^)				17.16 ± 0.05	20.22 ± 0.05	-	-
Voriconazole (200 µg.mL^−1^)				-	-	27.82 ± 0.30	41.14 ± 0.64
Sterile water				0.00	0.00	0.00	0.00

TL, Tasmannia lanceolata; DB, Diploglottis bracteata; SA, Syzygium aqueum.

**Table 3:** Soluble sugars, vitamin C, organic acids, and antioxidant capacity of aqueous extracts from *Tasmannia lanceolata* leaves, *Diploglottis bracteata* fruits, and *Syzygium aqueum* fruits.

		*T. lanceolata*	*D. bracteata*	*S. aqueum*
**Sugars (g. 100 g^−1^ dw)**	Fructose	2.08 ± 0.07 ^c^	17.15 ± 0.51 ^a^	10.52 ± 0.44 ^b^
	Glucose	1.79 ± 0.06 ^c^	15.12 ± 0.64 ^a^	6.33 ± 0.25 ^b^
	Sucrose	5.69 ± 0.08 ^a^	1.41 ± 0.07 ^b^	0.02 ± 0.00 ^c^
**Vitamin C (mg. 100 g^−1^ dw)**	L-AA	0.62 ± 0.01 ^b^	1.75 ± 0.02 ^a^	0.59 ± 0.01 ^b^
	Total AA	0.89 ± 0.02 ^c^	2.43 ± 0.03 ^a^	1.20 ± 0.10 ^b^
**Organic acids (g. 100 g^−1^ dw)**	Citric acid	1.43 ± 0.11 ^b^	1.75 ± 0.07 ^b^	28.81 ± 0.34 ^a^
	Fumaric acid	0.00 ± 0.00 ^b^	0.01 ± 0.00 ^a^	0.01 ± 0.00 ^a^
	Isocitric acid	0.36 ± 0.03 ^b^	0.37 ± 0.02 ^b^	0.49 ± 0.02 ^a^
	Malic acid	2.44 ± 0.21 ^c^	22.19 ± 0.11 ^a^	15.95 ± 0.43 ^b^
	Quinic acid	1.50 ± 0.11 ^b^	1.92 ± 0.08 ^b^	29.39 ± 0.28 ^a^
	Shikimic acid	5.30 ± 0.30 ^a^	0.01 ± 0.00 ^b^	0.09 ± 0.00 ^b^
	Succinic acid	0.02 ± 0.00 ^c^	0.10 ± 0.00 ^a^	0.06 ± 0.00 ^b^
**TPC (mg GAE.g^−1^ dw)**		123.47 ± 1.29 ^a^	6.07 ± 0.77 ^b^	6.45 ± 0.19 ^b^
**DPPH IC_50_ (μg.mL^−1^)**		36.59 ± 0.41 ^c^	353.60 ± 9.23 ^a^	299.89 ± 3.11 ^b^

Data are mean ± standard deviation (*n* = 3); data with different letters in the same row are significantly different (*p* < 0.05). GAE, gallic acid equivalents; dw, dry weight.

**Table 4:** Non-anthocyanin phenolic compounds (tentatively) identified in the aqueous extracts from *Tasmannia lanceolata* leaves.

Compound No.	RT (min)	[M-H]^−^ (m/z)	Molecular Formula	ΔM (ppm)	MS^2^ Fragmentation (m/z)	Tentative Identification
**Phenolic Acids**						
**1**	2.75	371.0984	C_16_H_20_O_10_	0.0808	371.0959; 191.0557; 135.0444; 85.0284	Hydroxydihydrocaffeoylquinic acid
**3**	3.87	315.0714	C_13_H_16_O_9_	−2.3994	315.0708; 108.0209; 152.0109; 207.9458	Protocatechuic acid O-hexoside
**4**	5.19	153.0189	C_7_H_6_O_4_	−2.8231	153.0189; 109.0286; 123.0445; 91.0173	Protocatechuic acid
**6**	6.40	371.0979	-	-	119.0496; 163.0394; 359.1305	p-coumaric acid derivative
**8**	6.64	707.1789	-	-	707.1731; 191.0559; 243.0657; 173.0452; 323.0540; 463.1036; 515.1109	Unknown, perhaps caffeoylquinic acid glucoside derivative
**10**	6.87	447.1867	-	-	153.0916; 137.0238; 271.0969; 359.0724	Unknown, perhaps hydroxybenzoic acid derivative
**12**	7.30	353.0864	C_16_H_18_O_9_	−3.9820	353.0857; 191.0556; 85.0284; 127.0392	Chlorogenic acid (syn: 5-caffeoylquinic acid) **
**13**	7.36	707.1799	C_32_H_36_O_18_	−4.2252	191.0556; 85.0284; 353.0851	Chlorogenic acid dimer
**14**	8.13	707.1787	C_32_H_36_O_18_	−5.9220	191.0556; 85.0284; 353.0832; 593.1383	Chlorogenic acid dimer isomer
**16**	8.40	337.0920	C_16_H_18_O_8_	−2.6431	337.0886; 93.0336; 119.0495; 173.0450; 163.0393; 87.0077; 255.1010	4-O-p-coumaroylquinic acid
**Flavonoids and derivatives**						
**15**	8.27	461.1658	-	-	101.0235; 113.0236; 289.0708; 153.0913; 161.0447; 329.1339	Unknown, perhaps tricin derivative
**26**	11.47	417.0824	-	-	417.0818; 284.0324; 315.0487; 133.0288	Unknown, luteolin derivative
**27**	11.47	547.1651	-	-	285.0395; 284.0325; 192.0422; 89.0233; 493.1665	Unknown, luteolin derivative
**32**	12.97	331.1208	-	-	331.1208; 96.9592; 219.1385; 263.1286; 269.0450	Unknown, perhaps apigenin derivative
**33**	13.07	505.2056	-	-	343.1535; 328.1302; 251.1653; 427.1900	Unknown, perhaps luteolin-trimethyl ester-O-hexoside
**34**	13.88	301.0349	C_15_H_10_O_7_	−1.5812	301.0337; 133.0290; 151.0033; 121.0290; 83.0128	Quercetin **
**35**	14.11	483.2434	-	-	299.0552; 284.0319; 209.0805; 165.0910	Unknown, perhaps diosmetin derivative
**36**	14.23	459.2218	-	-	96.9592; 331.1207; 299.0551; 284.0317	Unknown, perhaps diosmetin derivative
**37**	14.94	269.0449	C_15_H_10_O_5_	−2.4047	269.0453; 117.0339; 83.0128; 151.0030	Apigenin
**38**	16.27	299.0550	C_16_H_12_O_6_	−3.7183	299.0552; 284.0321; 133.0289; 203.1437; 107.0131; 168.9887; 256.0364; 265.1436; 83.0128	Diosmetin
**39**	17.29	283.0603	C_16_H_12_O_5_	−3.1689	283.0602; 117.0337; 268.0371; 237.1491; 211.0395; 107.0130; 151.0030; 239.0344; 191.1434; 83.0127	Apigenin 7,4′-dimethyl ether (syn: Genkwanin, Acacetin)
**Flavonoid glycosides**						
**17**	8.57	435.2226	-	-	289.0709; 177.0189; 339.0463; 245.0815	Unknown, perhaps catechin rhamnoside
**21**	9.94	609.1442	C_27_H_30_O_16_	−3.1322	609.1413; 300.0275; 447.0931; 151.0030	Rutin **
**22**	10.57	463.0879	C_21_H_20_O_12_	−0.6478	463.0865; 271.0243; 300.0266; 255.0293; 151.0029; 243.0294; 178.9978	Quercetin-3-O-glucoside **
**23**	11.05	863.1996	-	-	431.0974; 283.0605; 311.0549; 96.9591; 151.0030; 345.0993; 131.0495	Unknown, perhaps vitexin/isovitexin dimer
**24**	11.05	431.0981	C_21_H_20_O_10_	−0.6263	431.0975; 283.0606; 311.0549; 96.9591; 151.0030; 345.0996	Vitexin/isovitexin
**25**	11.25	593.1490	C_27_H_30_O_15_	−3.6988	593.1443; 285.0392; 255.0295; 361.1616; 165.0547; 523.2097	Kaempferol glycoside (perhaps Kaempferol O-hexosyl-deoxyhexose)
**28**	11.54	563.1383	C_26_H_28_O_14_	−4.1357	563.1362; 285.0397; 192.0423; 89.0232	Kaempferol glycoside (perhaps Kaempferol 3-O-rhamnoside-7-O-xyloside)
**30**	12.33	447.0929	C_21_H_20_O_11_	−0.8611	447.0861; 285.0402	Luteolin glycoside (perhaps Luteolin 8-C-glucoside)
**31**	12.43	593.1477	C_27_H_30_O_15_	−5.8905	269.0451; 547.1425	Apigenin dihexoside
**Biflavonoids & polyflavonoids**						
**2**	3.37	865.1781	-	-	140.0110; 287.0547; 407.0700; 543.0834; 451.0955	Perhaps procyanidin trimer (B-type)
**9**	6.77	577.1331	C_30_H_26_O_12_	−3.5520	577.1282; 125.0237; 289.0710; 161.0239; 245.0814; 407.0778; 205.0498	(epi)catechin-(epi)catechin OR procyanidin dimer (B type)
**18**	8.66	739.1623	C_39_H_32_O_15_	−6.1474	739.1487; 289.0714; 177.0191; 339.0488; 245.0814; 459.0654; 587.1086; 117.0551	Procyanidin dimer monoglycoside
**19**	8.75	577.1325	C_30_H_26_O_12_	−4.5916	125.0239; 289.0712; 245.0814; 491.01842; 203.0705	Procyanidin dimer (B type)
**20**	8.99	739.1622	C_39_H_32_O_15_	−6.2827	739.1502; 289.0712; 177.0190; 339.0489; 245.0813; 459.0662; 137.0239; 569.0995	Procyanidin dimer monoglycoside
**Other polyphenols**						
**5**	6.27	356.0976	-	-	121.0289; 237.403; 149.0603; 219.0293; 293.0631	Unknown, perhaps hydroxybenzaldehyde derivative
**7**	6.56	371.1336	-	-	243.0657; 323.0540; 289.0556; 173.0454	Unknown, perhaps piceatannol derivative
**11**	7.05	401.1434	-	-	401.1385; 96.9592; 361.0948; 134.0367; 239.0919; 271.0964	Unknown, perhaps pelargonidin-3-pentoside derivative
**29**	11.67	451.1031	C_24_H_20_O_9_	−0.7891	451.1018; 189.0189; 217.0136; 341.0650; 177.0188; 109.0287; 123.0444; 151.0395; 255.0294; 402.1244; 447.0855	Cinochonain l

RT, retention time. ** Commercial standard was used for identification.

**Table 5:** Non-anthocyanin phenolic compounds (tentatively) identified in the aqueous extracts from *Diploglottis bracteata* fruits.

Compound No.	RT (min)	[M-H]^−^ (m/z)	Molecular Formula	ΔM (ppm)	MS^2^ Fragmentation (m/z)	Tentative Identification
**Organic acids**						
**1**	1.39	133.0136	C_4_H_6_O_5_	−4.8641	133.0136; 115.0029; 89.0230; 111.0195; 124.0143	Malic acid
**2**	1.96	117.0185	-	-	117.0185; 100.0394	Unknown, perhaps succinic acid
**Phenolic acids**						
**14**	9.96	319.0790	-	-	119.0496	Unknown, perhaps coumaric acid derivative
**16**	11.14	355.1027	-	-	147.0445; 168.9885; 216.9797; 273.9771; 114.9481	Unknown, perhaps cinnamic acid derivative
**17**	11.80	415.1964	-	-	341.0607; 161.0609	Unknown, perhaps caffeoyl glucose derivative
**20**	12.51	631.2519	-	-	245.1545; 201.1647; 523.1197	Unknown, perhaps heptyl cinnamate derivative
**22**	14.62	509.2578	-	-	101.0235; 85.0284; 113.0239; 231.0989	Unknown, perhaps tetrahydrofurfuryl cinnamate derivative
**Flavonoids and derivatives**						
**8**	7.38	289.0713	C_15_H_14_O_6_	−1.5982	289.0710; 109.0287; 123.0444; 191.0556; 97.0286	Catechin **
**11**	8.15	417.1319	-	-	125.0239; 177.0190; 151.0396; 287.0552; 243.0294	Unknown, perhaps eriodictyol derivative
**12**	8.27	289.0707	C_15_H_14_O_6_	−3.6738	289.0710; 109.0286; 123.0444; 245.0814; 83.0127	Epicatechin **
**15**	10.91	495.1125	-	-	151.0034; 285.0397; 125.0236; 107.0130; 178.9980; 83.0128; 340.9699; 303.0478; 449.1015	Unknown, perhaps luteolin derivative
**21**	12.67	445.2071	-	-	445.1998; 165.1280; 209.1180; 283.1545; 337.0700; 87.0441	Unknown, perhaps wogonin derivative
**Flavonoid glycosides**						
**9**	7.82	447.1497	-	-	125.0236; 289.0712; 161.0238; 407.0796	Unknown, perhaps catechin glycoside
**18**	11.90	477.0980	-	-	477.0980; 299.0194; 119.0496; 314.0470	Perhaps isorhamnetin-3-O-hexoside
**Biflavonoids and polyflavonoids**						
**5**	6.30	593.1262	C_30_H_26_O_13_	−6.5145	177.0191; 339.0832; 273.0395	Prodelphinidin A-type
**6**	6.77	577.1323	C_30_H_26_O_12_	−4.9381	577.1257; 125.0238; 289.0714; 161.0240; 245.0814; 407.0787	Procyanidin dimer B-type
**7**	7.15	577.1317	C_30_H_26_O_12_	−5.9778	161.0241; 289.0709; 407.0772; 339.0813	Procyanidin dimer B-type
**10**	7.82	577.1317	C_30_H_26_O_12_	−5.9778	577.1245; 125.0236; 289.0712; 407.0786; 161.0238; 245.0813; 137.0237; 339.0815; 425.0938	Procyanidin dimer B-type
**13**	8.43	865.1772	-	-	165.0916; 287.0553; 543.0829; 577.1254	Perhaps procyanidin trimer/epicatechin-epicatechin-epicatechin
**Other polyphenols**						
**3**	3.16	344.1282	-	-	147.0441; 164.0703	Unknown, perhaps coumarin derivative
**4**	3.89	154.0505	-	-	82.0288; 108.0213	Unknown, perhaps hypogallic acid
**19**	12.43	489.2277	-	-	269.0455; 167.0343; 331.1887	Unknown, perhaps carnosic acid derivative

RT, retention time. ** Commercial standard was used for identification.

**Table 6:** Non-anthocyanin phenolic compounds (tentatively) identified in the aqueous extracts from *Syzygium aqueum* fruits.

Compound No.	RT (min)	[M-H]^−^ (m/z)	Molecular Formula	ΔM (ppm)	MS^2^ Fragmentation (m/z)	Tentative Identification
**Organic acid**						
**1**	1.46	133.0135	C_4_H_6_O_5_	−5.6159	133.0130; 111.0078; 115.0028; 96.9590; 107.2179	Malic acid
**2**	1.61	191.0189	C_6_H_8_O_7_	−4.3241	111.0078; 87.0076; 155.9504; 170.0026	Citric acid
**Phenolic acids**						
**3**	2.52	169.0132	C_7_H_6_O_5_	−6.1947	125.0236	Gallic acid **
**5**	6.87	327.0709	C_14_H_16_O_9_	−3.8401	312.045; 206.0210; 207.0290; 193.0130; 205.0137; 234.0162; 327.0663; 192.0060; 164.0109; 136.0159	Bergenin
**8**	7.53	759.1153	C_37_H_28_O_18_	−6.5707	759.1153; 175.0032; 289.0333; 301.0338; 423.0757; 345.0189; 481.0681; 468.0614	Theacitrin A
**Flavonoids and derivatives**						
**7**	7.30	511.1069	-	-	447.0876; 284.0318; 166.0265; 109.0288; 329.0816	Unknown, perhaps kaempferol derivative
**11**	8.12	305.0691	-	-	96.9592; 125.0238; 169.0137; 177.0188; 243.0294; 305.0660	Unknown, perhaps gallocatechin gallate
**26**	12.39	317.0290	C_15_H_10_O_8_	−4.0721	301.0347; 151.0031; 109.0287; 137.0238; 178.9981; 227.343	Myricetin **
**27**	13.88	301.0342	C_15_H_10_O_7_	−3.9065	151.0031; 107.0130; 121.0288; 93.0036; 83.0128	Quercetin **
**Flavonoid glycosides**						
**6**	7.30	447.0927	C_21_H_20_O_11_	−1.3084	447.0082; 285.0385; 241.0499; 147.0081; 199.0394; 329.0816	Luteolin-3-glucoside
**14**	9.00	479.0811	C_21_H_20_O_13_	−4.2038	479.0776; 316.0215; 271.0242; 287.0191; 372.9598; 214.0260	Myricetin-3-O-β-D-galactopyranoside isomer
**15**	9.11	479.0807	C_21_H_20_O_13_	−5.0387	479.0750; 316.0220; 271.0250; 287.0190; 109.0290	Myricetin-3-O-β-D-galactopyranoside
**17**	10.03	449.0719	-	-	449.0633; 316.0214; 271.0246; 287.0185; 283.0604; 242.0224; 405.9114; 214.0263	Kamepferol derivative; perhaps dihydrokaempferol-hexoside
**18**	10.20	431.0970	C_21_H_20_O_10_	−3.1779	283.0605; 311.0553; 341.0697; 323.0517; 239.0716; 211.0756	Vitexin or isovitexin
**20**	10.41	597.1743	-	-	597.1736; 357.0947; 387.1089; 209.0447; 239.0576; 417.1104	Perhaps phloretin-di-glucoside
**21**	10.55	463.0862	C_21_H_20_O_12_	−4.3188	463.0862; 300.0268; 271.0245; 255.0295; 151.0031; 356.9632; 390.9250	Quercetin-3-O-glucoside **
**22**	10.83	479.0798	C_21_H_20_O_13_	−6.9173	479.0728; 178.9982; 317.0288; 406.9325	Myricetin-glycoside
**23**	11.10	433.0806	C_20_H_18_O_11_	6.8463	433.0674; 300.0269; 301.0339; 271.0248; 255.0287; 243.0299; 390.9264; 356.9643	Quercetin-glycoside
**24**	11.33	435.1340	C_21_H_24_O_10_	9.9487	125.0240; 167.0340; 273.0750; 315.0840; 369.0010	Phloridzin
**25**	11.53	433.0766	C_20_H_18_O_11_	−2.3898	433.0691; 271.0246; 300.0246; 315.0123; 163.0029; 299.9917	Quercetin-glycoside
**Tannins**						
**4**	3.67	933.0374	-	-	933.0371; 300.9981; 275.0193; 125.0238; 229.0140; 314.0032; 421.0121; 467.0170; 492.9950; 569.0468; 613.0347; 871.0412	Perhaps castalagin
**9**	7.61	1139.3467	-	-	177.0189; 125.0237; 169.0139; 243.0297; 759.1021; 633.0806	Unknown, perhaps galloylated tannin
**10**	7.68	953.0593	-	-	953.0590; 125.0240; 177.0190; 169.0140; 165.0190; 137.0240; 151.0400; 243.0300; 275.0180; 301.0320; 299.0160; 423.0650; 759.1010; 935.0510; 633.0800	Perhaps chebulagic acid
**12**	8.52	911.1115	-	-	911.1061; 125.0239; 169.0137; 96.9592; 177.0188; 137.0238; 285.0399; 571.0787; 741.0919; 423.0636; 615.0666; 305.0635	Perhaps theasinesin A
**13**	8.66	935.0502	-	-	935.0495; 300.9984; 125.0238; 169.0138; 275.0186; 633.0611	Perhaps casuarinin
**16**	9.43	895.1121	-	-	895.1100; 299.9907; 447.0488; 361.1577; 300.9964; 555.0822	Unknown, ellagic acid derivative
**19**	10.33	300.9978	C_14_H_6_O_8_	−3.9568	300.9978; 145.0287; 169.0133; 117.0338; 245.0082; 283.9944; 228.0052; 200.0106	Ellagic acid **

RT, retention time. ** Commercial standard was used for identification.

**Table 7:** ANOVA for the determination of model fitting (inhibition zone of extract blends).

Parameters		Box–Behnken Design				Simplex–Lattice Design			
		*PV*	*BS*	*RD*	*AA*	*PV*	*BS*	*RD*	*AA*
**Model**	df	6	9	7	6	5	4	4	4
	F-value	428.33	53.74	59.88	13.06	85.69	7.43	431.83	19.38
	*p*-value	<0.0001	<0.0001	<0.0001	<0.0003	<0.0001	<0.0063	<0.0001	<0.0002
**Residual**	df	10	7	9	10	8	9	9	9
	msq	1.55	0.7169	3.19	0.7455	1.03	1.71	0.0246	0.0836
	ssq	15.54	5.02	28.70	7.45	8.25	15.39	0.2212	0.7527
**Lack of fit**	df	6	3	5	6	4	5	5	5
	F-value	1.81	2.51	15.49	5.95	2.51	1.51	7.63	0.7315
	*p*-value	0.2937	0.1980	0.0100	0.0531	0.1971	0.3558	0.0357	0.6365
**Pure error**	df	4	4	4	4	4	4	4	4
	msq	1.04	0.4357	0.3524	0.1879	0.5872	1.33	0.0052	0.0983
	ssq	4.18	1.74	1.41	0.7516	2.35	5.34	0.0210	0.3932
**R^2^**		0.9961	0.9857	0.9790	0.8869	0.9817	0.7675	0.9948	0.8960
**R_a_^2^**		0.9938	0.9674	0.9626	0.8190	0.9702	0.6641	0.9925	0.8497

*PV*, *Pseudomonas viridiflava*; *BS*, *Bacillus subtilis*; *RD*, *Rhodotorula diobovata*; *AA*, *Alternaria alternate*; df, degrees of freedom; *p*-values < 0.05 were significant; msq, mean square; ssq, sum of squares; R^2^, coefficient of determination; R_a_^2^, adjusted coefficient of determination.

**Table 8:** Validation of predicted and experimental values for the inhibitory activity (mm) of the two optimised extract blends (*n* = 5).

Response	Predicted Mean Value	Experimental Value	−95% Prediction	+95% Prediction
**Box–Behnken**				
*Pseudomonas viridiflava*	17.21	16.78	16.78	17.64
*Bacillus subtilis*	15.90	14.54	14.52	17.29
*Rhodotorula diobovata*	25.29	26.34	22.49	28.09
*Alternaria alternata*	5.29	6.31	4.03	6.55
**Simplex–Lattice**				
*Pseudomonas viridiflava*	15.12	14.53	12.93	17.32
*Bacillus subtilis*	14.03	12.95	11.33	16.72
*Rhodotorula diobovata*	20.41	22.84	17.53	23.44
*Alternaria alternata*	4.42	6.96	2.07	7.31

## Data Availability

The data presented in this study are available on request from the corresponding author.

## Supplementary Materials

The following supporting information can be downloaded at: https://www.mdpi.com/article/10.3390/foods12030623/s1, Representative UHPLC-UV chromatograms (Figure S1), mass spectral data of non-identified compounds in the extracts (Tables S1–S3) and of commercial standards (Table S4), full-MS scan and product ion mass spectra of selected identified compounds (Figures S2–S7), ANOVA (Tables S5–S12) and normal plot of residuals and predicted versus actual plot (Figures S8–S15) for the activity against studied microorganisms are summarized in the Supplementary Materials.
