# Supplementary material for: Valorisation of Three Underutilised Native Australian Plants: Phenolic and Organic Acid Profiles and In Vitro Antimicrobial Activity
Source: Foods. 2023 Feb 1;12(3):623. doi: 10.3390/foods12030623 (PMC9914099; doi:10.3390/foods12030623)
Supplement: Supplementary file 1 [file foods-12-00623-s001.zip › foods-2153702-supplementary.pdf]

Supplementary Material

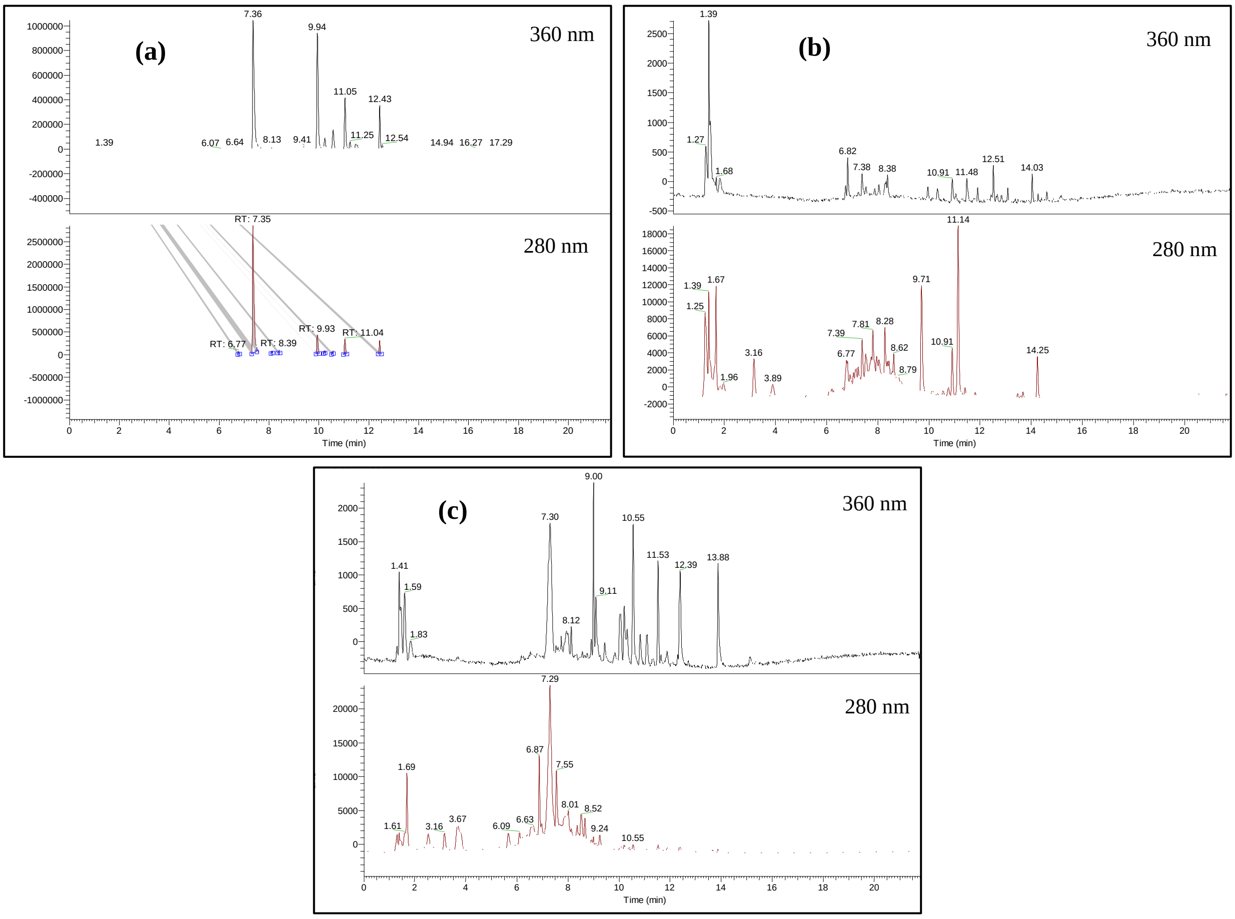

Figure S1. Representative UHPLC-UV chromatograms of (a) *Tasmannia lanceolata*, (b) *Diploglottis bracteata*, and (c) *Syzygium aqueum* water extracts at 280 and 360 nm.

Table S1. Unknown phenolic compounds in the water extract of *Tasmannia lanceolata* leaves characterized by HRAM-UHPLC-Q/Orbitrap-MS/MS in negative ion mode.

No.	UV RT (min)	$[M-H]^-$ (m/z)	MS^2 Product Ions (m/z)
**1**	5.00	331.1025	135.0444; 85.0285
**2**	5.69	395.1913	177.0553; 149.0604; 161.0603; 109.0287; 283.0837
**3**	6.19	461.1292	461.1229; 152.0111; 135.0449; 271.0970
**4**	6.53	345.1181	133.0290; 161.0244; 293.0095
**5**	7.19	361.0956	361.0949; 96.9592; 150.0316; 194.0211; 209.0446
**6**	7.68	595.1268	595.1203; 299.0187; 491.1847; 432.0618; 355.0767; 149.0604
**7**	7.82	315.1078	125.0237; 145.0289; 96.9592; 289.0711
**8**	7.82	489.1961	145.0289; 101.0236; 443.1847; 281.1375
**9**	7.87	373.1467	145.0290; 125.0238; 96.9593; 117.0339; 345.0983; 289.0707
**10**	8.01	355.1025	160.0162; 132.0212; 175.0396; 259.0603
**11**	8.01	439.1817	101.0236; 205.0710
**12**	8.49	345.1006	345.0998; 96.9592; 135.0445; 269.1744
**13**	9.11	441.1971	165.0551; 311.0543; 150.0316; 101.0238
**14**	9.11	593.1468	893.1408; 311.0549; 165.0551; 101.0237
**15**	9.14	337.0918	337.0885; 179.0709; 85.0286; 93.0338; 275.1642; 127.0398
**16**	9.23	319.0451	109.0288; 151.0395; 183.0290; 97.0286; 287.0543
**17**	9.41	475.1813	327.0500; 96.9592; 361.1638; 101.0236; 115.0394; 131.0344; 143.0342; 165.0549; 205.0708; 421.1788
**18**	9.83	345.1001	345.0999; 96.9592; 135.0446; 285.0398; 317.1033
**19**	10.23	563.1383	563.1376; 311.0553; 283.0606; 427.2029
**20**	11.75	497.1074	177.0189; 341.0650; 109.0287; 161.0239; 281.0439; 326.1107
**21**	12.43	329.1050	329.1048; 96.9591; 275.1632; 135.0443
**22**	12.54	401.0873	401.0880; 268.0377; 240.0422
**23**	12.80	459.2228	101.0236; 85.0285; 113.0237; 161.0449; 413.2223; 277.1803
**24**	12.97	471.1864	263.1286; 219.1385; 96.9592; 331.1206; 203.1070
**25**	13.20	459.2231	96.9592; 331.1204; 269.1749
**26**	13.32	329.1055	329.1051; 96.9592; 219.0689
**27**	13.51	447.0724	447.0762; 295.0244; 219.0662; 135.0446; 355.1127
**28**	13.94	317.1419	317.1417; 193.0864; 271.1002
**29**	13.94	507.2429	115.0394; 131.0344; 143.0343; 327.0860; 205.0704
**30**	14.38	643.3035	109.0290; 305.1943; 515.2770; 242.0568
**31**	15.52	265.1440	265.1440; 221.1535; 175.1123; 177.1278; 161.0965; 191.1434; 205.1229; 109.0652; 97.0285; 147.0806
**32**	19.16	116.9277	99.9249; 83.9298; 100.9254; 112.9850
**33**	19.69	540.3268	255.2324; 224.0683; 168.0427

Table S2. Unknown phenolic compounds in the water extract of *Diploglottis bracteata* fruits characterized by HRAM-UHPLC-Q/Orbitrap-MS/MS in negative ion mode.

No.	UV RT (min)	$[M\text{-}H]^-$ (m/z)	MS^2 Product Ions (m/z)
**1**	1.68	249.0248	249.0239; 128.0347; 216.9429
**2**	1.83	304.9902	304.9898; 133.0135; 115.0028; 96.9687; 298.8889
**3**	5.23	299.0767	92.0253; 251.9612; 229.0750; 198.0424
**4**	6.10	203.0824	116.0501
**5**	6.21	342.0864	134.0468; 107.0357; 189.9807; 233.9709; 223.9413
**6**	6.38	505.1847	153.0912; 297.1304
**7**	6.64	281.1237	156.9583; 263.1076; 118.9920; 276.0995
**8**	6.74	293.1235	245.0817; 113.0602; 237.1484
**9**	6.81	444.1646	444.4553; 136.0399; 92.0496; 221.0915; 402.1343
**10**	6.92	305.0659	305.0647; 137.0240; 109.0288; 93.0337; 139.0396; 165.0189; 219.0652
**11**	7.06	413.1660	189.1278; 251.1280; 381.1966; 295.1151
**12**	7.23	220.0974	220.0978; 117.0340; 89.0382
**13**	7.53	325.0918	325.0878; 117.0339; 145.0290; 287.0555; 167.0345
**14**	7.66	427.1820	101.0235; 85.0284; 113.0236; 168.9882; 235.1176
**15**	8.04	519.2064	165.0917; 217.1062
**16**	8.27	461.1658	165.0914; 101.0235; 289.0707; 85.0284
**17**	8.38	519.2058	165.0916; 325.0336; 487.0818
**18**	8.62	401.0870	401.0808; 151.0396; 109.0288; 137.0239; 203.0710
**19**	8.71	277.1285	277.1291; 235.1183; 168.9888; 257.0996; 148.9088
**20**	8.71	323.1334	235.1183; 168.9888; 161.0447; 291.0531; 257.0996
**21**	8.71	375.1650	235.1183; 168.9888; 291.0518; 355.0845
**22**	8.79	521.2219	521.2164; 101.0235; 143.0343; 205.0710; 89.0233; 319.0074; 385.0909; 511.1870
**23**	9.71	865.1759	865.1764; 227.0710; 261.0404; 589.0918; 177.0552
**24**	9.90	311.1124	311.1117; 269.1029; 168.9888; 161.0449; 251.0915; 114.9484
**25**	9.90	357.1181	269.1028; 168.9888; 291.0840; 325.0346; 148.09091
**26**	9.90	711.2048	269.1028; 168.9888; 161.0449; 80.9158; 148.9091; 291.0841; 301.0322; 592.1174; 358.9947
**27**	10.33	301.1758	301.1738; 128.0349; 271.0240; 84.0442
**28**	10.33	639.1452	639.1440; 316.0210; 331.0419; 271.0240
**29**	11.05	321.1541	141.0913; 95.3013; 247.9760
**30**	11.05	547.2366	89.0234; 99.0079; 191.0553; 149.0450; 459.0798

Table S2. *Continued.*

No.	UV RT (min)	$[M\text{-}H]^-$ (m/z)	MS^2 Product Ions (m/z)
**31**	11.48	455.2129	168.9888; 101.0235; 113.0236; 85.0284; 263.1487
**32**	11.48	415.1965	168.9888; 101.0235
**33**	12.04	417.2120	152.0111; 168.9889; 283.1534; 229.1436; 325.1699
**34**	13.08	351.1647	319.0811; 289.1421; 221.1529
**35**	13.47	609.1736	255.0661; 121.0288; 563.1646
**36**	13.47	581.1781	121.0289; 148.0526; 227.0698; 279.0643
**37**	13.57	529.2577	305.2124
**38**	13.67	263.1285	122.0367; 203.1071; 143.1070; 253.0525; 188.0832
**39**	14.03	483.2434	101.0235; 113.0236; 168.9886; 347.1106; 291.1791; 85.0285
**40**	14.27	425.1732	219.1014; 147.0439; 289.1428
**41**	14.33	351.1646	143.1067
**42**	15.15	186.1030	186.1024; 170.0731
**43**	15.90	511.2732	101.0235; 85.0284

Table S3. Unknown phenolic compounds in the water extract of *Syzygium aqueum* fruits characterized by HRAM-UHPLC-Q/Orbitrap-MS/MS in negative ion mode.

No.	UV RT (min)	$[M-H]^-$ (m/z)	MS^2 Product Ions (m/z)
**1**	6.09	203.0822	116.0500; 95.9752; 142.0655; 203.0820; 127.9435
**2**	6.39	285.0612	285.0590; 108.0210; 125.0238; 177.0190; 119.0496; 96.9593; 207.9453; 241.9102
**3**	6.63	475.0325	367.0038
**4**	7.30	935.0509	935.0545; 284.0318; 285.0385; 166.0265; 125.0235; 149.0237; 147.0081; 241.0499; 255.0294; 217.0500; 199.0394; 329.0820; 447.0875; 465.0947; 917.0438; 519.0116
**5**	7.61	531.0835	125.0237; 177.0187; 169.0136; 137.0237; 243.0295; 301.0340; 423.0760
**6**	7.74	387.1676	387.1600; 125.0239; 177.0191; 169.0139; 243.0296; 149.0240; 355.0766; 269.0447; 111.0084; 268.0371; 345.0772
**7**	7.93	479.1180	479.1092; 299.0545; 298.0474; 283.0244; 284.0312; 284.0312; 149.0238; 165.0188; 177.0189; 137.0238; 317.0616; 423.0624; 461.1016
**8**	8.12	305.0691	96.9592; 125.0238; 169.0137; 177.0188; 243.0294; 305.0660
**9**	8.37	245.0118	165.0189; 183.0294; 139.0394
**10**	8.91	436.0762	357.0924; 327.0463; 387.1142; 417.1259; 121.0291; 152.0107; 295.1164
**11**	9.24	319.0446	97.0285; 139.0393; 153.0187; 115.0392; 208.9536
**12**	11.66	477.0961	447.0988; 255.0297; 227.0344; 125.0236; 284.0306; 169.0138; 177.0184; 327.0812
**13**	11.88	479.1115	151.0033; 271.0610; 119.0496; 344.0466
**14**	13.49	549.3326	137.0238; 165.0187; 503.3309
**15**	13.67	263.1283	122.0365; 203.1069; 136.0522; 188.0832
**16**	15.13	186.1030	128.0377; 186.1026
**17**	16.40	305.0807	108.0210; 211.0392; 183.0436; 247.0756

Table S4. Retention time and mass spectra data of commercial standards analysed by HRAM-UHPLC-Q/Orbitrap-MS/MS in negative ion mode.

No.	UV RT (min)	$[M-H]^-$ (m/z)	MS^2 Product Ions (m/z)	Compound
**1**	2.53	169.0130	169.0136; 125.0237; 119.0493; 117.0337; 91.0542	Gallic acid
**2**	7.37	289.0708	289.0707; 109.0285; 123.0442; 125.0235; 245.0812; 137.0236; 151.0392; 203.0705; 97.0285; 205.0496; 159.0443; 83.0127; 179.0338; 221.0807; 168.9873; 85.0285	Catechin
**3**	7.37	353.0868	353.0810; 191.0555; 85.0284; 93.0336; 127.0393; 135.0444; 87.0077; 280.9485; 248.0839	Chlorogenic acid
**4**	7.99	179.0342	179.0340; 135.0442; 134.0366; 89.0386; 136.0475; 108.0210; 117.0339; 168.9885	Caffeic acid
**5**	8.27	289.0708	289.0707; 109.0285; 123.0442; 137.0236; 125.0235; 97.0285; 151.0393; 245.0812; 95.0492; 203.0704; 159.0443; 83.0127; 93.0334	Epicatechin
**6**	9.56	163.0393	163.0392; 93.0335; 119.0493; 117.0337; 91.542	p-Coumaric acid
**7**	9.95	609.1428	609.1401; 300.0271; 301.0330; 426.9658	Rutin
**8**	10.27	300.9979	300.9978; 145.0288; 173.0236; 200.0107; 117.0337; 283.9954; 185.0236; 163.0393; 216.0056; 161.0237; 157.0288; 133.0288; 245.0080	Ellagic acid
**9**	10.55	463.0874	463.0859; 300.0268; 271.0243; 255.0293; 301.0321; 151.0029; 243.0293; 178.9978; 272.0282;	Quercetin-3-glucoside
**10**	12.38	317.0291	317.0288; 109.0286; 137.0237; 151.0030; 107.0130; 83.0127; 178.0998; 315.0134; 125.0236; 287.0181; 271.0236	Myricetin
**11**	13.84	285.0397	285.0396; 133.0287; 132.0211; 107.0129; 134.0319; 83.0127; 151.0029; 175.0390; 121.0286; 199.0389; 241.0486	Luteolin
**12**	13.87	301.0344	301.0342; 151.0030; 107.0129; 93.0335; 121.0287; 178.9979; 139.0393; 168.9885	Quercetin
**13**	15.10	285.0396	285.0396; 93.0335; 117.0337; 143.0495; 159.0444; 108.0208; 145.0287; 227.0339; 211.0390; 255.0291; 131.0495; 183.0439; 267.0269	Kaempferol

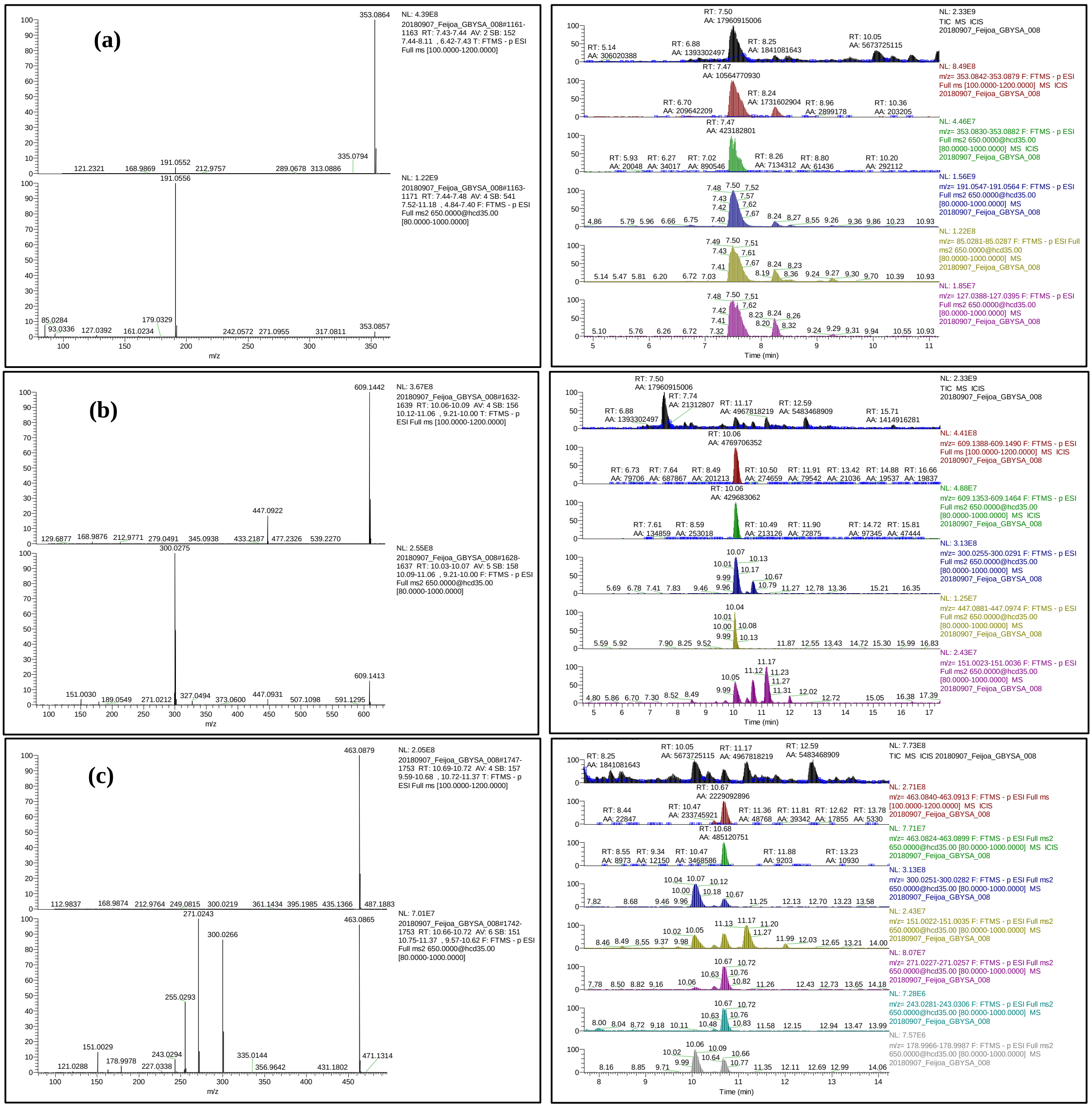

Figure S2. Full-MS scan (m/z 100 – 1200) and product ion mass spectra of (a) chlorogenic acid (m/z 353.0864), (b) rutin (m/z 609.1442), and (c) quercetin-3-O-glucoside (m/z 463.0879) in *Tasmannia lanceolata* water extract by HRAM-UHPLC-Q/Orbitrap-MS/MS in negative ion mode.

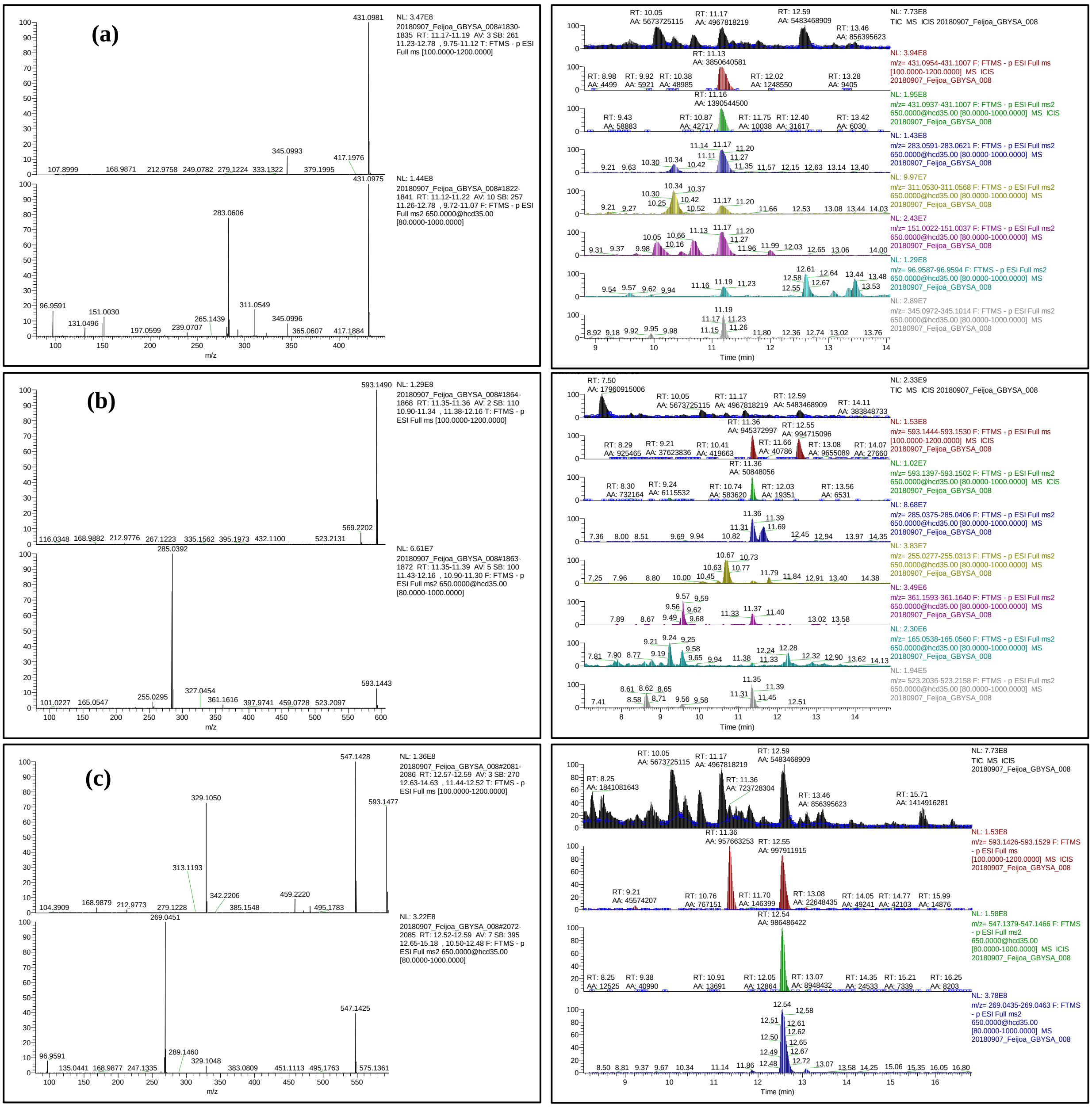

Figure S3. Full-MS scan (m/z 100 – 1200) and product ion mass spectra of (a) vitexin/isovitexin (m/z 431.0981), (b) kaempferol glycoside (m/z 593.1490), and (c) apigenin dihexoside (m/z 593.1477) in *Tasmannia lanceolata* water extract by HRAM-UHPLC-Q/Orbitrap-MS/MS in negative ion mode.

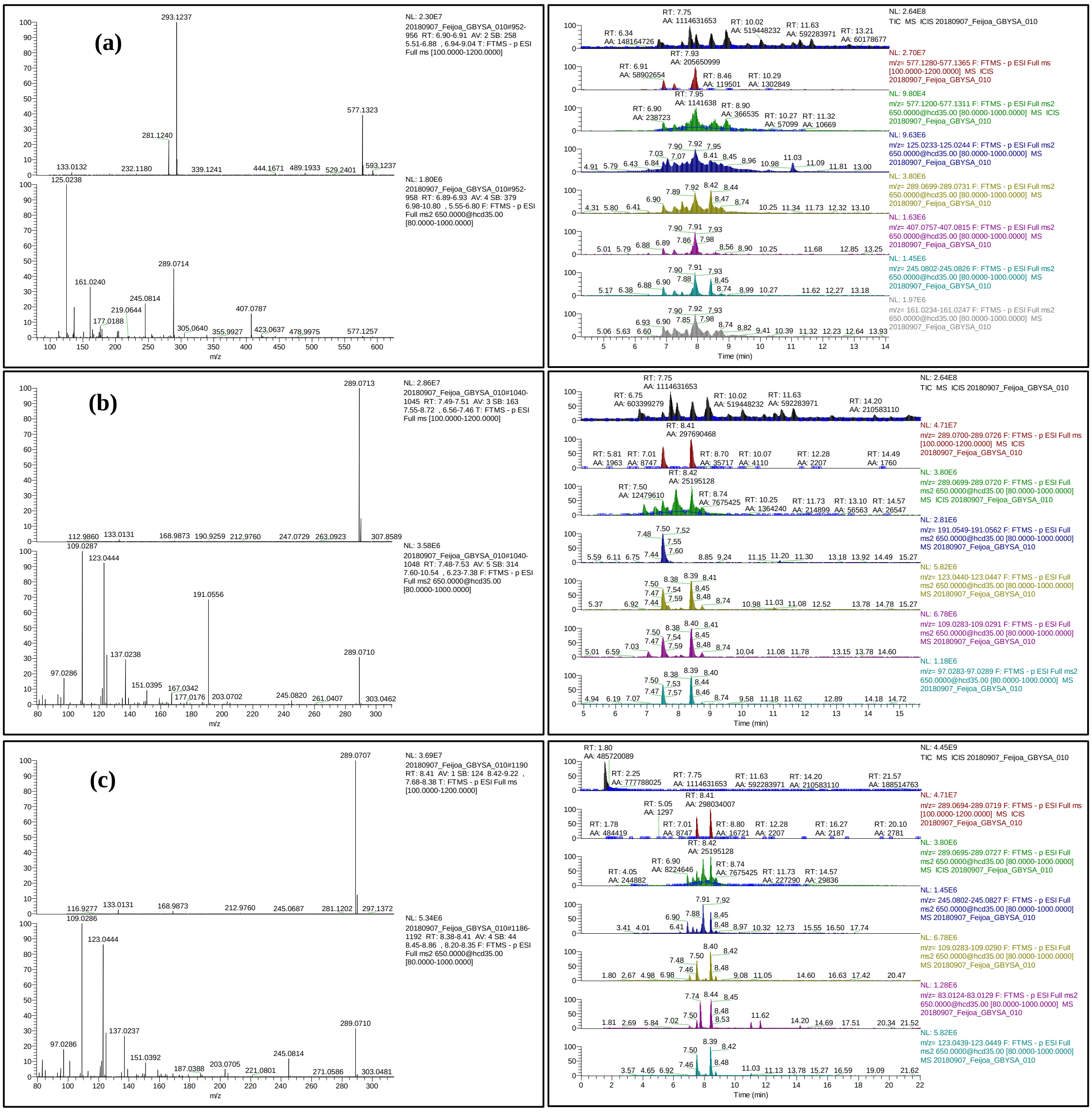

Figure S4. Full-MS scan (m/z 100 – 1200) and product ion mass spectra of (a) B-type procyanidin dimer (m/z 577.1323), (b) catechin (m/z 289.0713), and (c) epicatechin (m/z 289.0707) in *Diploglottis bracteata* water extract by HRAM-UHPLC-Q/Orbitrap-MS/MS in negative ion mode.

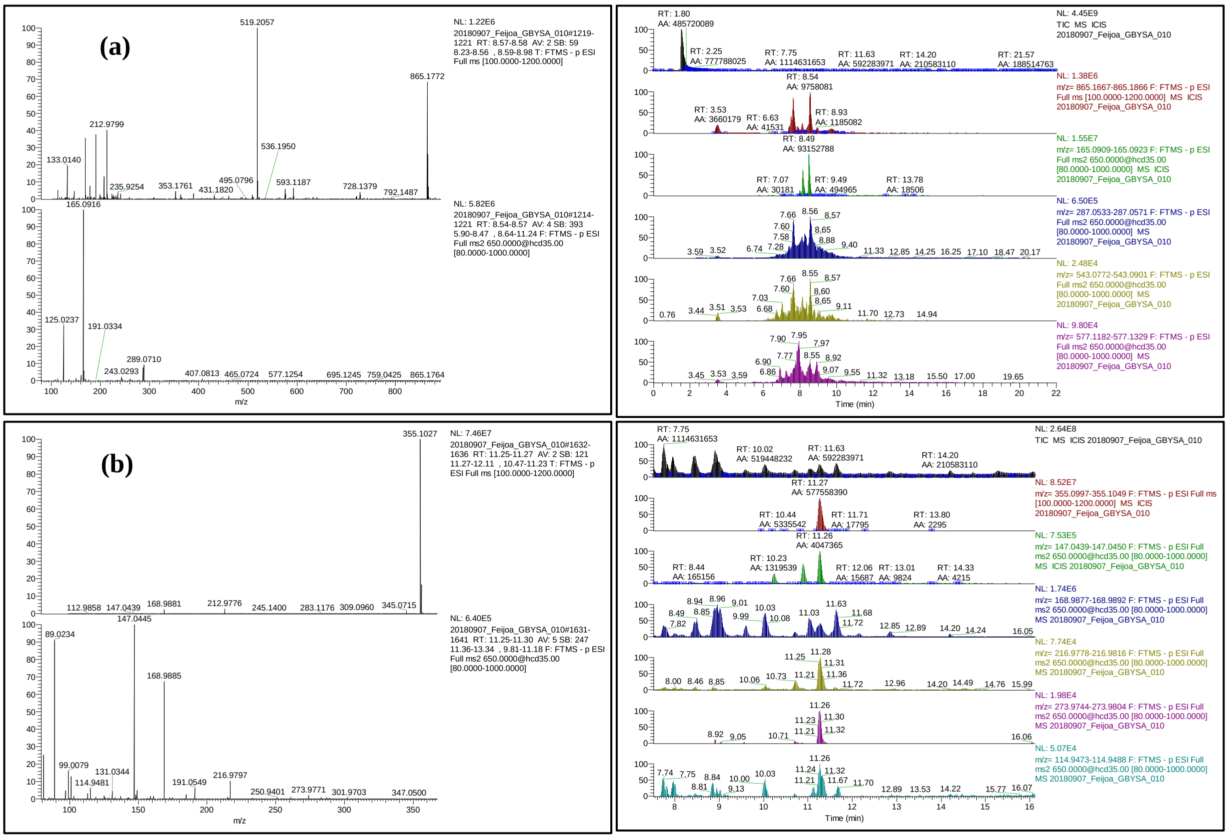

Figure S5. Full-MS scan (m/z 100 – 1200) and product ion mass spectra of (a) (most likely) procyanidin trimer (m/z 865.1772) and (b) (most likely) cinnamic acid derivative (m/z 355.1027) in *Diploglottis bracteata* water extract by HRAM-UHPLC-Q/Orbitrap-MS/MS in negative ion mode.

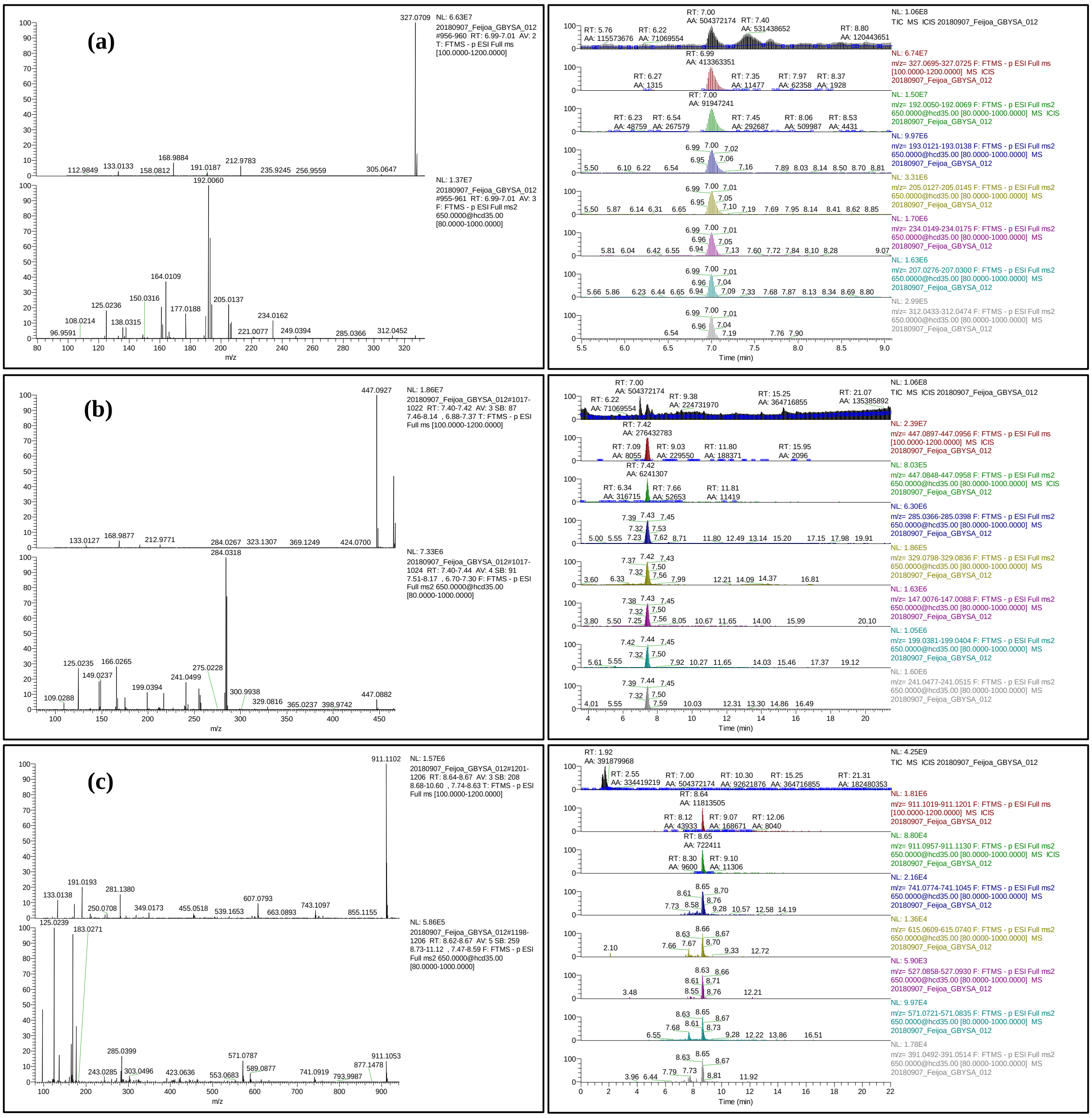

Figure S6. Full-MS scan (m/z 100 – 1200) and product ion mass spectra of (a) bergenin (m/z 327.0709), (b) cyanidin-3-glucoside (m/z 447.0927), and (c) theasinesin A (m/z 911.1061) in *Syzygium aqueum* water extract by HRAM-UHPLC-Q/Orbitrap-MS/MS in negative ion mode.

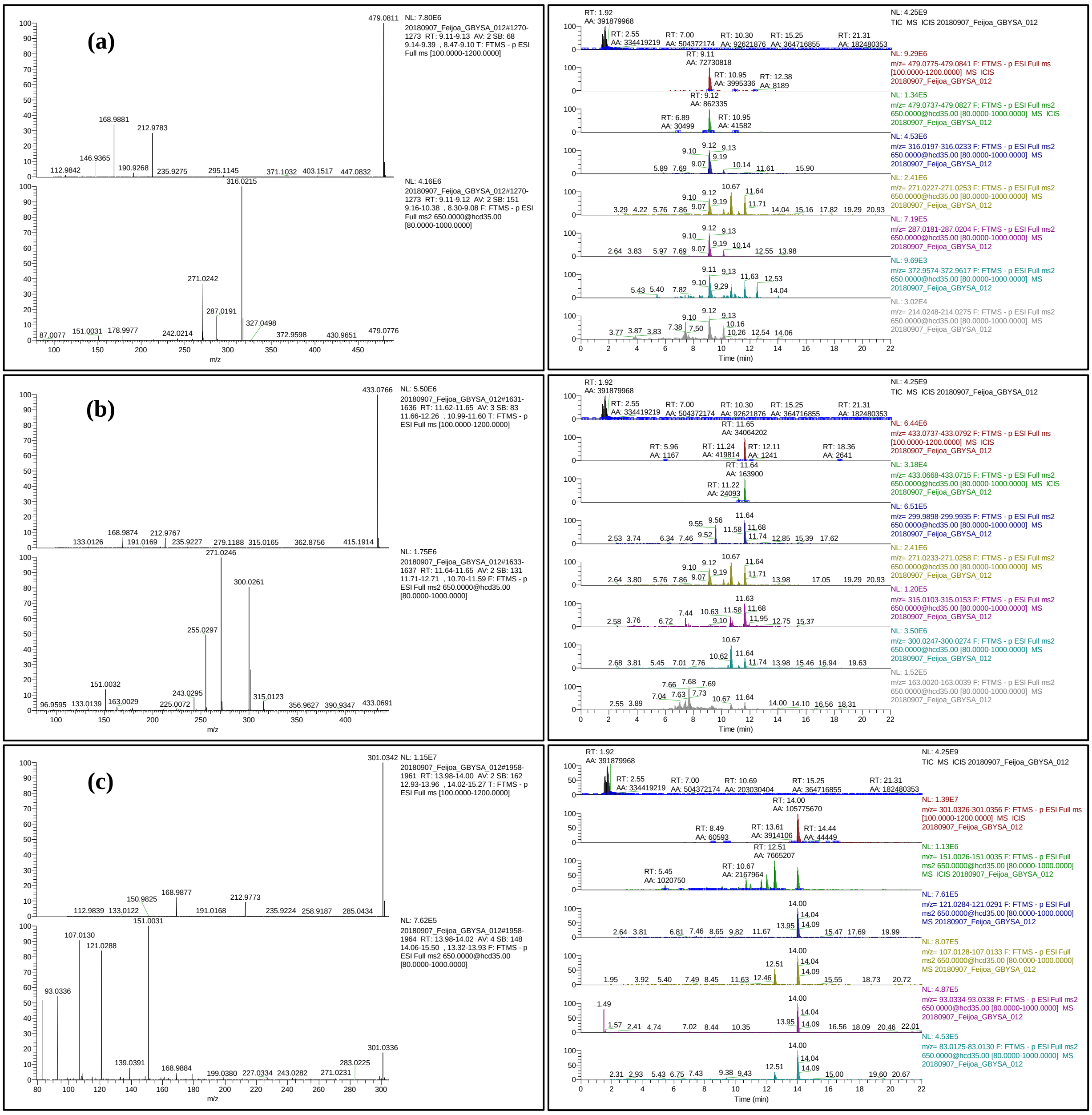

Figure S7. Full-MS scan (m/z 100 – 1200) and product ion mass spectra of (a) myricetin-3-O-β-galactopyranoside (m/z 479.0811), (b) quercetin glycoside (m/z 433.0766), and (c) quercetin (m/z 301.0342) in *Syzygium aqueum* water extract by HRAM-UHPLC-Q/Orbitrap-MS/MS in negative ion mode.

Table S5. ANOVA for the inhibition zone (mm) of the extracts blend against *Pseudomonas viridiflava* (reduced quadratic model, power transformed (lambda: 1.35; constant: 0); Box-Behnken design).

Source	Sum of Squares	df	Mean Square	F-value	p-value	
**Model**	3992.50	6	665.42	428.33	< 0.0001	significant
**A-TPL**	0.0354	1	0.0354	0.0228	0.8830	
**B-BT**	849.06	1	849.06	546.54	< 0.0001	
**C-LP**	2638.45	1	2638.45	1698.35	< 0.0001	
**BC**	220.16	1	220.16	141.72	< 0.0001	
**B^2**	63.25	1	63.25	40.72	< 0.0001	
**C^2**	207.92	1	207.92	133.84	< 0.0001	
**Residual**	15.54	10	1.55			
**Lack of Fit**	11.36	6	1.89	1.81	0.2937	not significant
**Pure Error**	4.18	4	1.04			
**Cor Total**	4008.04	16				
Fit Statistics						
**Std. Dev.**	**Mean**	**C.V. (%)**	**R^2**	**Adjusted R^2**	**Predicted R^2**	**Adeq Precision**
1.25	41.47	3.01	0.9961	0.9938	0.9858	72.4915

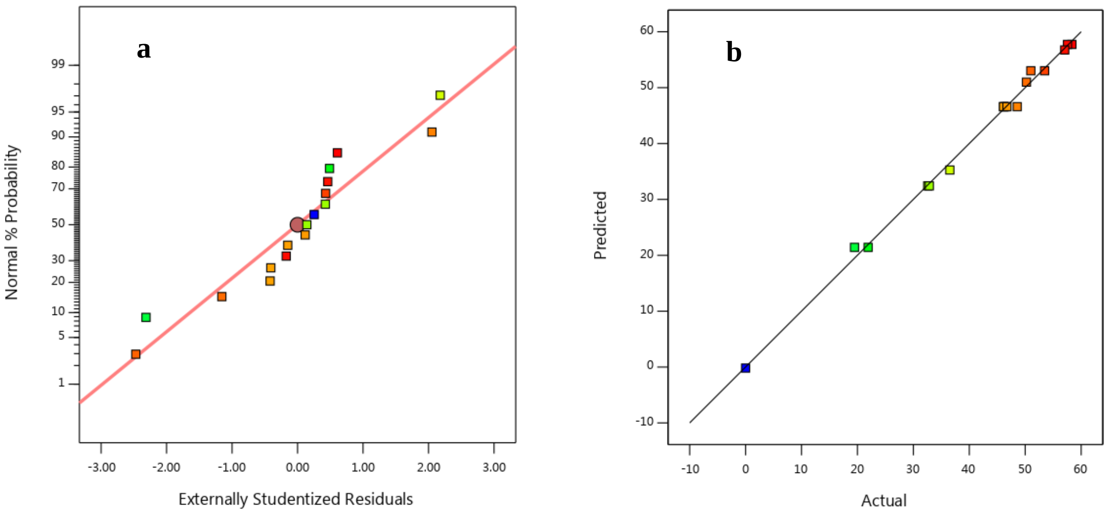

Figure S8. Normal plot of residuals (a) and predicted versus actual (b) of the inhibition zone of extract blends against *Pseudomonas viridiflava* (power transformed, lambda 1.35). Colour points by value of Inhibition zone of extracts solution, 0.000 58.355.

Table S6. ANOVA for the inhibition zone (mm) of the extracts blend against *Bacillus subtilis* (quadratic model; Box-Behnken design).

Source	Sum of Squares	df	Mean Square	F-value	p-value	
**Model**	346.70	9	38.52	53.74	< 0.0001	significant
**A-TPL**	107.36	1	107.36	149.76	< 0.0001	
**B-BT**	18.63	1	18.63	25.98	0.0014	
**C-LP**	124.35	1	124.35	173.46	< 0.0001	
**AB**	4.33	1	4.33	6.04	0.0436	
**AC**	10.84	1	10.84	15.11	0.0060	
**BC**	15.85	1	15.85	22.12	0.0022	
**A^2**	46.12	1	46.12	64.34	< 0.0001	
**B^2**	1.40	1	1.40	1.95	0.2051	
**C^2**	13.31	1	13.31	18.57	0.0035	
**Residual**	5.02	7	0.7169			
**Lack of Fit**	3.28	3	1.09	2.51	0.1980	not significant
**Pure Error**	1.74	4	0.4357			
**Cor Total**	351.71	16				
Fit Statistics						
**Std. Dev.**	**Mean**	**C.V. (%)**	**R^2**	**Adjusted R^2**	**Predicted R^2**	**Adeq Precision**
0.8467	12.56	6.74	0.9857	0.9674	0.8433	25.2435

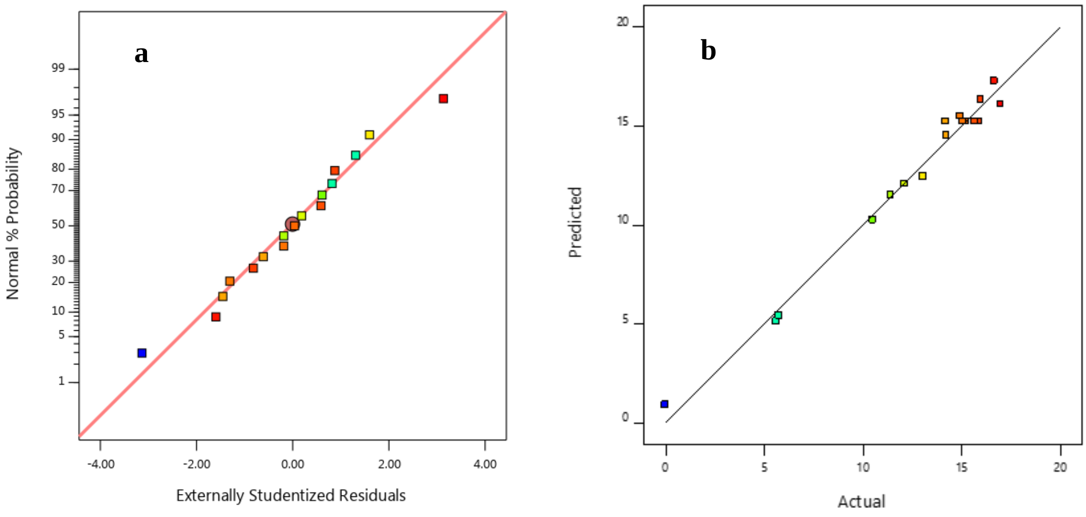

Figure S9. Normal plot of residuals (a) and predicted versus actual (b) of the inhibition zone of extract blends against *Bacillus subtilis*. Colour points by value of Inhibition zone of extracts solution, 0 16.9767 .

Table S7. ANOVA for the inhibition zone (mm) of the extracts blend against *Rhodotorula diobovata* (reduced quadratic model; Box-Behnken design).

Source	Sum of Squares	df	Mean Square	F-value	p-value	
**Model**	1336.33	7	190.90	59.88	< 0.0001	significant
**A-TPL**	1059.23	1	1059.23	332.22	< 0.0001	
**B-BT**	6.37	1	6.37	2.00	0.1913	
**C-LP**	1.97	1	1.97	0.6179	0.4520	
**BC**	31.72	1	31.72	9.95	0.0117	
**A^2**	179.33	1	179.33	56.25	< 0.0001	
**B^2**	16.77	1	16.77	5.26	0.0475	
**C^2**	23.96	1	23.96	7.51	0.0228	
**Residual**	28.70	9	3.19			
**Lack of Fit**	27.29	5	5.46	15.49	0.0100	significant
**Pure Error**	1.41	4	0.3524			
**Cor Total**	1365.02	16				
Fit Statistics						
**Std. Dev.**	**Mean**	**C.V. (%)**	**R^2**	**Adjusted R^2**	**Predicted R^2**	**Adeq Precision**
1.79	15.09	11.83	0.9790	0.9626	0.8103	20.2444

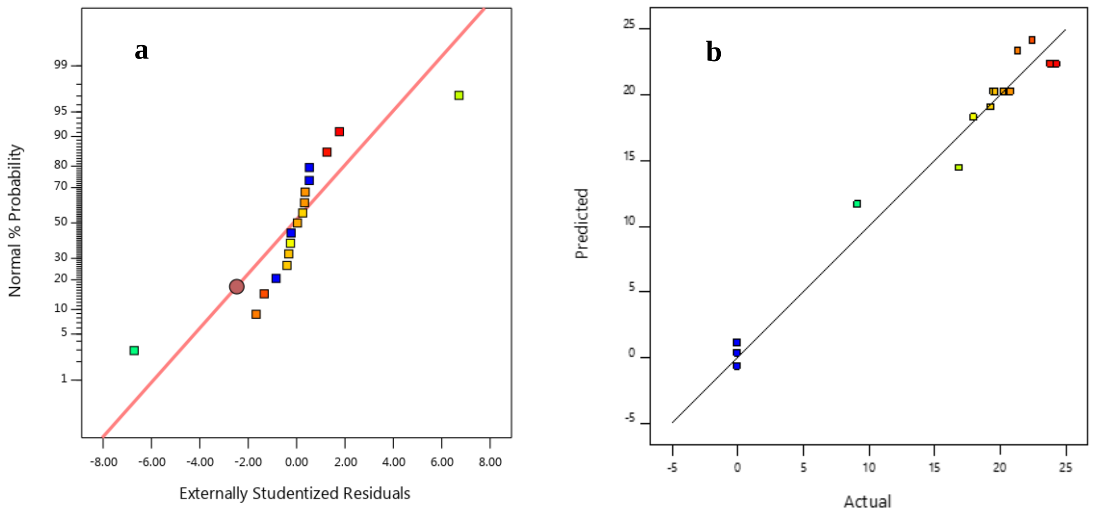

Figure S10. Normal plot of residuals (a) and predicted versus actual (b) of the inhibition zone of extract blends against *Rhodotorula diobovata*. Colour points by value of Inhibition zone of extracts solution, 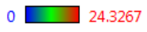

.

Table S8. ANOVA for the inhibition zone (mm) of the extracts blend against *Alternaria alternata* (reduced quadratic model; Box-Behnken design).

Source	Sum of Squares	df	Mean Square	F-value	p-value	
**Model**	58.43	6	9.74	13.06	0.0003	significant
**A-TPL**	37.83	1	37.83	50.75	< 0.0001	
**B-BT**	0.0011	1	0.0011	0.0015	0.9703	
**C-LP**	3.81	1	3.81	5.12	0.0472	
**BC**	8.97	1	8.97	12.03	0.0060	
**A^2**	3.20	1	3.20	4.29	0.0653	
**B^2**	5.04	1	5.04	6.76	0.0265	
**Residual**	7.45	10	0.7455			
**Lack of Fit**	6.70	6	1.12	5.95	0.0531	not significant
**Pure Error**	0.7516	4	0.1879			
**Cor Total**	65.88	16				
Fit Statistics						
**Std. Dev.**	**Mean**	**C.V. (%)**	**R^2**	**Adjusted R^2**	**Predicted R^2**	**Adeq Precision**
0.8634	2.64	32.73	0.8869	0.8190	0.5613	12.6414

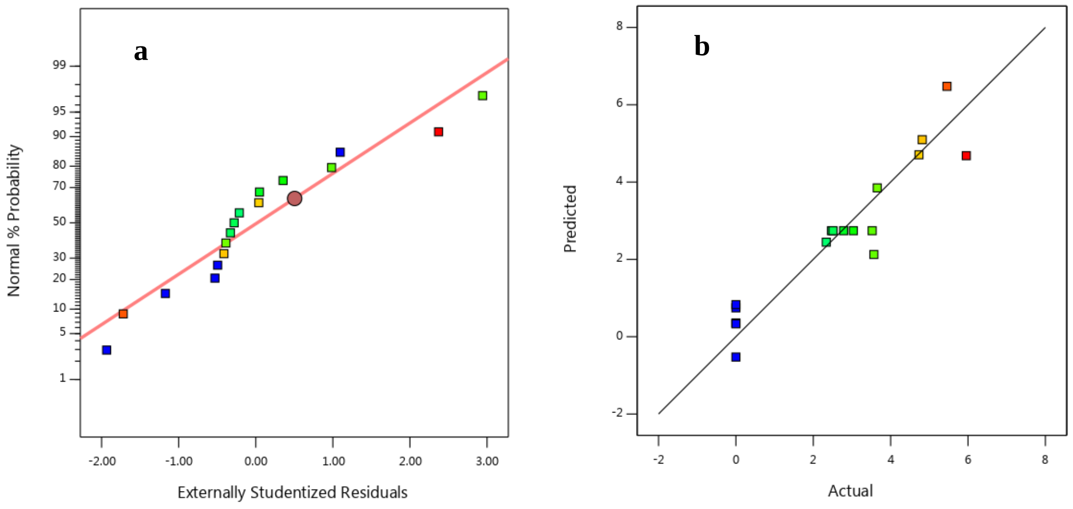

Figure S11. Normal plot of residuals (a) and predicted versus actual (b) of the inhibition zone of extract blends against *Alternaria alternata*. Colour points by value of Inhibition zone of extracts solution, 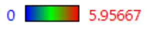

.

Table S9. ANOVA for the inhibition zone (mm) of the extracts blend against *Pseudomonas viridiflava* (quadratic model; mixture design).

Source	Sum of Squares	df	Mean Square	F-value	p-value	
**Model**	441.73	5	88.35	85.69	< 0.0001	significant
**(1) Linear Mixture**	401.72	2	200.86	194.83	< 0.0001	
**AB**	12.79	1	12.79	12.41	0.0078	
**AC**	27.03	1	27.03	26.22	0.0009	
**BC**	0.0535	1	0.0535	0.0519	0.8255	
**Residual**	8.25	8	1.03			
**Lack of Fit**	5.90	4	1.47	2.51	0.1971	not significant
**Pure Error**	2.35	4	0.5872			
**Cor Total**	449.97	13				
Fit Statistics						
**Std. Dev.**	**Mean**	**C.V. (%)**	**R^2**	**Adjusted R^2**	**Predicted R^2**	**Adeq Precision**
1.02	12.10	8.39	0.9817	0.9702	0.9509	27.6336

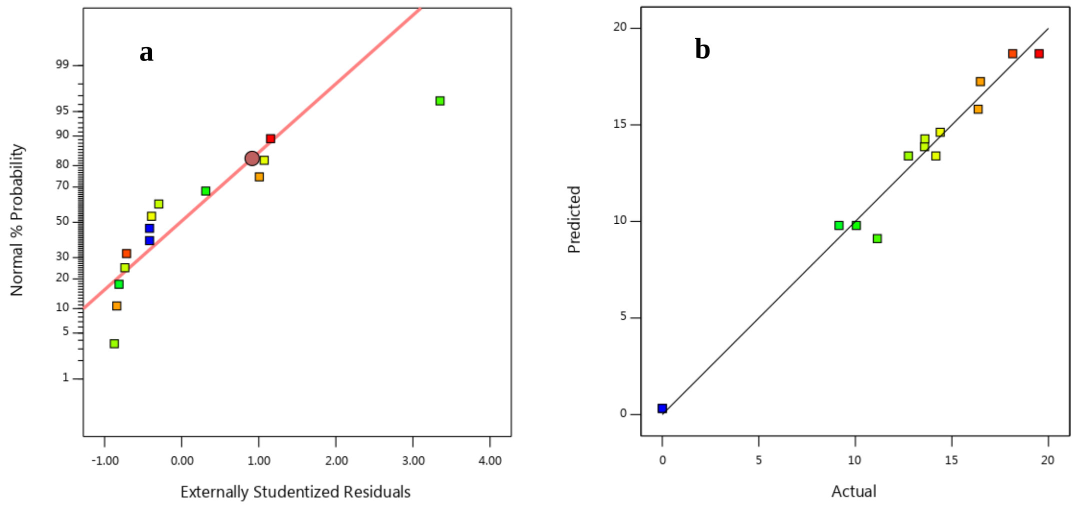

Figure S12. Normal plot of residuals (a) and predicted versus actual (b) of the inhibition zone of extract blends against *Pseudomonas viridiflava* (mixture design). Colour points by value of Inhibition zone of extracts solution, 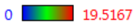

.

Table S10. ANOVA for the inhibition zone (mm) of the extracts blend against *Bacillus subtilis* (reduced quadratic model; mixture design).

Source	Sum of Squares	df	Mean Square	F-value	p-value	
**Model**	50.81	4	12.70	7.43	0.0063	significant
**[1]Linear Mixture**	25.17	2	12.58	7.36	0.0128	
**AB**	13.90	1	13.90	8.13	0.0191	
**AC**	11.69	1	11.69	6.83	0.0281	
**Residual**	15.39	9	1.71			
**Lack of Fit**	10.06	5	2.01	1.51	0.3558	not significant
**Pure Error**	5.34	4	1.33			
**Cor Total**	66.20	13				
Fit Statistics						
**Std. Dev.**	**Mean**	**C.V. (%)**	**R^2**	**Adjusted R^2**	**Predicted R^2**	**Adeq Precision**
1.31	10.77	12.15	0.7675	0.6641	0.5153	8.5557

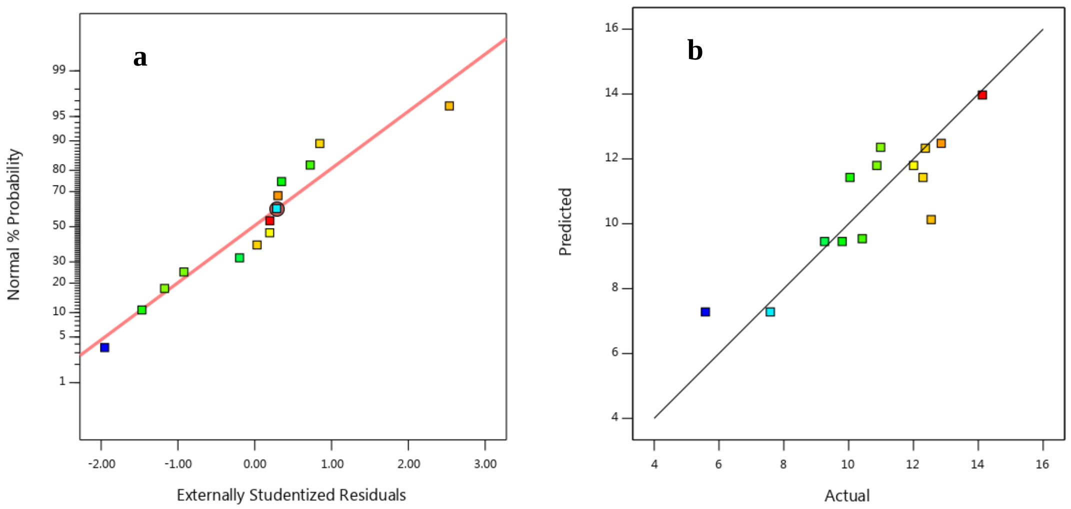

Figure S13. Normal plot of residuals (a) and predicted versus actual (b) of the inhibition zone of extract blends against *Bacillus subtilis* (mixture design). Colour points by value of Inhibition zone of extracts solution, 5.58 14.1267 .

Table S11. ANOVA for the inhibition zone (mm) of the extracts blend against *Rhodotorula diobovata* (reduced quadratic model, square root transformed, constant 0.5; mixture design).

Source	Sum of Squares	df	Mean Square	F-value	p-value	
**Model**	42.45	4	10.61	431.83	< 0.0001	significant
**(1)Linear Mixture**	33.17	2	16.59	674.84	< 0.0001	
**AB**	4.81	1	4.81	195.87	< 0.0001	
**AC**	4.45	1	4.45	180.95	< 0.0001	
**Residual**	0.2212	9	0.0246			
**Lack of Fit**	0.2002	5	0.0400	7.63	0.0357	significant
**Pure Error**	0.0210	4	0.0052			
**Cor Total**	42.67	13				
Fit Statistics						
**Std. Dev.**	**Mean**	**C.V. (%)**	**R^2**	**Adjusted R^2**	**Predicted R^2**	**Adeq Precision**
0.1568	2.84	5.52	0.9948	0.9925	0.9906	45.6061

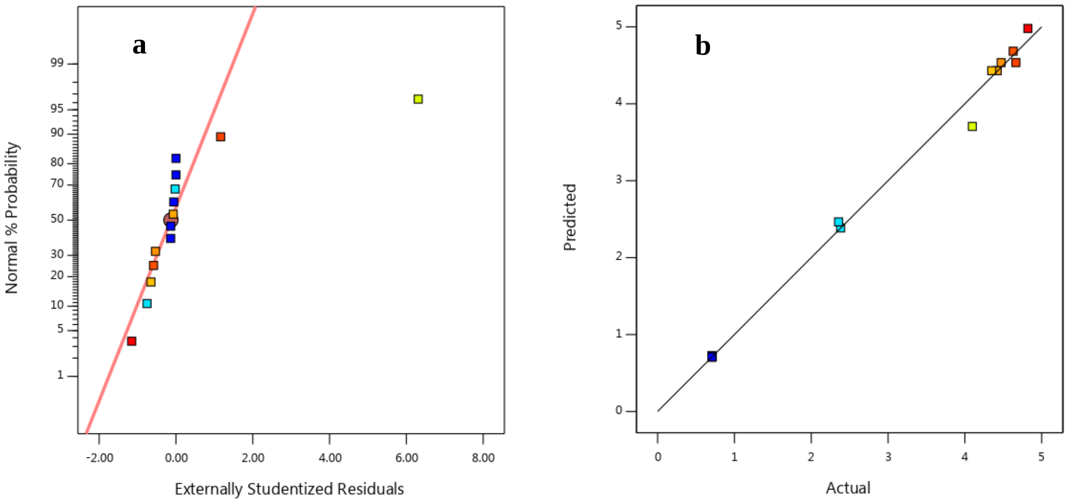

Figure S14. Normal plot of residuals (a) and predicted versus actual (b) of the inhibition zone of extract blends against *Rhodotorula diobovata* (mixture design). Colour points by value of square root of (inhibition zone of extracts solution + 0.5), 0.707 4.820 .

Table S12. ANOVA for the inhibition zone (mm) of the extracts blend against *Alternaria alternata* (reduced quadratic model, square root transformed, constant 0.5; mixture design).

Source	Sum of Squares	df	Mean Square	F-value	p-value	
**Model**	6.48	4	1.62	19.38	0.0002	significant
**(1)Linear Mixture**	5.19	2	2.60	31.05	< 0.0001	
**AB**	0.9964	1	0.9964	11.91	0.0073	
**AC**	0.2903	1	0.2903	3.47	0.0954	
**Residual**	0.7527	9	0.0836			
**Lack of Fit**	0.3595	5	0.0719	0.7315	0.6365	not significant
**Pure Error**	0.3932	4	0.0983			
**Cor Total**	7.24	13				
Fit Statistics						
**Std. Dev.**	**Mean**	**C.V. (%)**	**R^2**	**Adjusted R^2**	**Predicted R^2**	**Adeq Precision**
0.2892	1.67	17.33	0.8960	0.8497	0.7116	10.4282

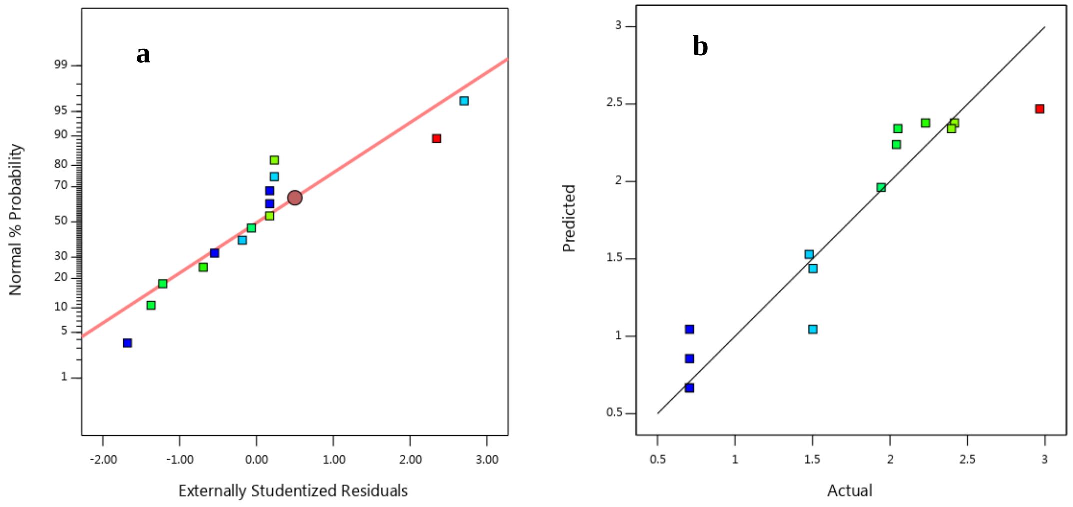

Figure S15. Normal plot of residuals (a) and predicted versus actual (b) of the inhibition zone of extract blends against *Alternaria alternata* (mixture design). Colour points by value of square root of (inhibition zone of extracts solution + 0.5), 0.707 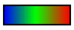 2.966
